# Targeting surface nucleolin with multivalent HB-19 and related Nucant pseudopeptides results in distinct inhibitory mechanisms depending on the malignant tumor cell type

**DOI:** 10.1186/1471-2407-11-333

**Published:** 2011-08-03

**Authors:** Bernard Krust, Diala El Khoury, Isabelle Nondier, Calaiselvy Soundaramourty, Ara G Hovanessian

**Affiliations:** 1 CNRS-Université Paris Descartes, Unité Régulation de la Transcription de Maladies Génétique, 45 rue des Saints Pères, 75270 Paris Cedex 06, France

**Keywords:** antitumoral action, surface nucleolin, multivalent pseudopeptides, nucleolin antagonist peptide, anti-inflammatory action, nucleophosmin

## Abstract

**Background:**

Nucleolin expressed at the cell surface is a binding protein for a variety of ligands implicated in tumorigenesis and angiogenesis. By using a specific antagonist that binds the C-terminal RGG domain of nucleolin, the HB-19 pseudopeptide, we recently reported that targeting surface nucleolin with HB-19 suppresses progression of established human breast tumor cells in the athymic nude mice, and delays development of spontaneous melanoma in the RET transgenic mice.

**Methods:**

By the capacity of HB-19 to bind stably surface nucleolin, we purified and identified nucleolin partners at the cell surface. HB-19 and related multivalent Nucant pseudopeptides, that present pentavalently or hexavalently the tripeptide Lysψ(CH_2_N)-Pro-Arg, were then used to show that targeting surface nucleolin results in distinct inhibitory mechanisms on breast, prostate, colon carcinoma and leukemia cells.

**Results:**

Surface nucleolin exists in a 500-kDa protein complex including several other proteins, which we identified by microsequencing as two Wnt related proteins, Ku86 autoantigen, signal recognition particle subunits SRP68/72, the receptor for complement component gC1q-R, and ribosomal proteins S4/S6. Interestingly, some of the surface-nucleolin associated proteins are implicated in cell signaling, tumor cell adhesion, migration, invasion, cell death, autoimmunity, and bacterial infections. Surface nucleolin in the 500-kDa complex is highly stable. Surface nucleolin antagonists, HB-19 and related multivalent Nucant pseudopeptides, exert distinct inhibitory mechanisms depending on the malignant tumor cell type. For example, in epithelial tumor cells they inhibit cell adhesion or spreading and induce reversion of the malignant phenotype (BMC cancer 2010, **10**:325) while in leukemia cells they trigger a rapid cell death associated with DNA fragmentation. The fact that these pseudopeptides do not cause cell death in epithelial tumor cells indicates that cell death in leukemia cells is triggered by a specific signaling mechanism, rather than nonspecific cellular injury.

**Conclusions:**

Our results suggest that targeting surface nucleolin could change the organization of the 500-kDa complex to interfere with the proper functioning of surface nucleolin and the associated proteins, and thus lead to distinct inhibitory mechanisms. Consequently, HB-19 and related Nucant pseudopeptides provide novel therapeutic opportunities in treatment of a wide variety of cancers and related malignancies.

## Background

Nucleolin is a multifunctional DNA-, RNA- and protein-binding protein ubiquitously expressed in exponentially growing eukaryotic cells. It is involved in fundamental aspects of transcription, cell proliferation and growth [1,2]. Nucleolin is found at several locations in cells: in the nucleolus it controls many aspects of DNA and RNA metabolism [3]; in the cytoplasm it shuttles proteins into the nucleus and provides a post-transcriptional regulation of strategic mRNAs [4,5]; and on the cell surface it serves as an attachment protein for several ligands from growth factors to microorganisms [6-12]. In contrast to nuclear nucleolin, surface nucleolin is glycosylated and is constantly induced in proliferating tumor and endothelial cells [6,13-15].

Surface nucleolin serves as a low affinity receptor for HIV-1 and various growth factors that interact with its C-terminal domain containing nine repeats of the tripeptide arginine-glycine-glycine, known as the RGG or GAR domain [10,16-20]. Binding of these ligands results in clustering of cell-surface nucleolin in lipid raft membrane microdomains before endocytosis of the ligand-nucleolin complex [10,17,19]. Accordingly, surface nucleolin could shuttle ligands between the cell surface and the nucleus thus act as a mediator for the extracellular regulation of nuclear events [18,20,21]. Moreover, ligand binding to surface nucleolin could generate high transitory intracellular Ca^2+ ^membrane fluxes, and thus initiate signal transduction events [13,22-25]. For an example, the binding of P-selectin to human colon carcinoma cells is shown to induce tyrosine phosphorylation of surface nucleolin and formation of a signaling complex containing nucleolin, phosphatidylinositol 3-kinase (PI3-K) and p38 MAPK [26].

The importance of cell-surface nucleolin in cancer biology was recently highlighted by studies showing that ligands of nucleolin play critical role in tumorigenesis and angiogenesis [20,26-36]. Accordingly, we recently reported that both of these events are suppressed by targeting surface nucleolin with the HB-19 pseudopeptide, a potent antagonist that forms an irreversible complex with surface nucleolin [9,37]. By binding to the RGG domain of nucleolin, HB-19 prevents binding of growth factors to cells, triggers calcium entry into cells, inhibits MAP kinase activation, and down-regulates surface nucleolin without affecting nuclear nucleolin [7,9,13,16,18,19,37]. In nude mice, we showed that HB-19 treatment markedly suppresses the progression of established human breast tumor cell xenografts, and in some cases eliminates measurable tumors [37]. This potent antitumoral effect *in vivo *is attributed to the direct dual inhibitory action of HB-19 on tumor and endothelial cells [37]. In a more relevant tumor model, we showed that HB-19 treatment for several months delays significantly the onset and frequency of spontaneous melanoma in RET mice, impairs tumor angiogenesis, and reduces metastasis while displaying no toxicity to normal tissue [38]. Other groups have reported that guanosine-rich quadruplex-forming oligodeoxynucleotides (GROs), which interact with surface nucleolin and/or intracellular nucleolin are promising agents for treatment of cancer [39-41]. The aptamer AS1411 is the most recent GRO that is currently being tested in Phase II clinical trials. Finally, we recently reported that treatment of G401 cells derived from a rhabdoid tumor of the kidney and TIII cells derived from a malignant melanoma can affect several criteria implicated in their tumorigenic potential, such as restoration of contact inhibition, reduction of colony formation in soft agar, and impairment of tumorigenicity in mice [38,42]. Interestingly, these changes are associated with a selective down regulation of genes implicated in tumorigenesis.

Although nucleolin does not possess a hydrophobic transmembrane domain to account for its expression at the cell surface, it behaves as a typical membrane-anchored receptor as demonstrated by its clustering when intact cells are incubated with the anti-nucleolin monoclonal antibody. This clustering occurs at the external side of the plasma membrane and is dependent on its indirect association with the intracellular actin cytoskeleton [6]. An actin based motor protein, the nonmuscle myosin heavy chain 9, could serve as a physical linker between surface nucleolin and actin [34]. Here we report that surface nucleolin exists in a high molecular weight complex, referred to as 500-kDa complex, in association with proteins partners known for their implication in tumorigenesis, inflammation, and bacterial infections. As the 500-kDa complex is highly stable, targeting surface nucleolin could change the organization of this complex and thus interfere with the proper functioning of surface nucleolin and the associated proteins. Indeed, by using HB-19 and related multivalent Nucant pseudopeptides that present pentavalently or hexavalently the tripeptide Lysψ(CH_2_N)-Pro-Arg (Nucant 3, 6, 6L and 7) [43,44], we show that such surface nucleolin antagonists exert distinct inhibitory mechanisms depending on the malignant tumor cell type. Accordingly, they inhibit the production of pro-inflammatory cytokines by human blood lymphocytes in response to stimulation with inactivated *Staphyloccocus aureus*, inhibit adhesion of human breast and prostate carcinoma cells, impair spreading of human colon carcinoma cells, and induce a selective cell killing in leukemia cells. Our results further validate surface nucleolin as a strategic target for an effective cancer drug.

## Methods

### Cells and culture medium

Human breast (MDA-MB-231, MDA-MB-435 [37]), prostate (LNCaP, from American Type Culture Collection, ATCC, Rockville, MD), and cervical (HeLa [45]) epithelial cancer cells were grown in DMEM-glutamax medium (Gibco Invitrogen, Cergy-Pontoise, France) containing GlutaMAX™, 4.5 g/l glucose and supplemented with 10% heat-inactivated (56°C for 30 min) fetal bovine serum (FBS; Hyclone, Thermo Fisher Scientific, Inc). Human SW480 cells are derived from a grade 3-4 colon carcinoma, while the highly metastatic counterpart SW620 is established from the lymph node of a 51-year-old Caucasian male. Both SW480 and SW620 cells (obtained from Francis Raul, IRCAD) are highly tumorigenic in nude mice. They were cultured in D-MEM, 10% FCS. The murine melanoma TIII cell line was obtained from Armelle Prévost-Blondel [38]. Murine T29 lymphoma cells, obtained from Philippe Kastner (CNRS, Strasbourg), have been established from Ik^L/L ^tumors from mice carrying a hypomorphic mutation (Ik^L/L^) in the Ikaros gene encoding a transcription factor that regulates lymphocyte differentiation, and acts also as a tumor suppressor in T lymphocytes [46]. The T29 cells were cultured in RPMI medium containing 25 mM Hepes pH 7.6, 1 mM sodium pyruvate, and 10% FCS. Human leukemia cell lines, Jurkat (acute T cell leukemia), HuT 78 (cutaneous T lymphocyte), RAJI (Burkitt lymphoma), and HL60 (promyelocytic leukemia) were cultured in RPMI-1640 medium (Gibco) containing GlutaMAX™, and supplemented with 10% heat-inactivated FBS as described before [15]. The wild-type Chinese hamster ovary cell line (CHO K1) was purchased from American Type Culture Collection (ATCC; Rockville, MD). The CHO LRP1-null cell line (CHO-13-5-1) was kindly provided by D. Strickland [47]. They were cultured in Ham's F12K medium. All cells were cultured with 10% (v/v) heat inactivated (56°C, 30 min) fetal bovine serum (FBS) (Roche Molecular Biochemicals, Indianapolis, IN) and 50 international units/mL penicillin-streptomycin (Invitrogen). Cell death was monitored conveniently by uptake of the trypan blue dye due to plasma membrane permeability of dying and/or dead cells.

### Peptide constructs

The HB-19 pseudopeptide 5[Kψ(CH_2_N)PR-TASP, for [Lysψ(CH_2_N)Pro-Arg]-template-assembled synthetic peptide, binds specifically surface nucleolin and blocks its function [9,16,37]. The template in HB-19, presents pentavalently the tripeptide 5[Kψ(CH_2_N)PR where (CH_2_N) represents a reduced peptide bond between lysine and proline residues. The synthesis of HB-19 and biotinylated HB-19 (HB-19/Btn) used for the recovery of surface nucleolin were as described previously [16,48]. Other peptide constructs similar to HB-19 are referred to as Nucant for Nucleolin antagonist. They present the pseudo-tripeptide Lysψ(CH_2_N)-Pro-Arg pentavalently (N3) or hexavalently (N6, N6L, N7) as described (Table 1; Additional file 1, Figure S1) [43,44,49]. N3, N6, N7, and the biotinylated N3 and N7 were synthesized by Jean Paul Briand, IBMC-CNRS, Strasbourg [43]. N6L was kindly provided by R. Zimmer (ImmuPharma). All peptides were obtained at a high purity (95%). They are all readily soluble in distilled water or PBS.

**Table 1:** HB-19 and related Nucant pseudopeptides.

**HB-19**

Presents pentavalently the pseudo-tripeptide Lysψ(CH_2_N)-Pro-Arg coupled to the template: H_2_N**Lys**-Lys-**Lys**-Gly-Pro-**Lys**-Glu-**Lys**-AhxCONH_2_.

**Nucant 7: N7**

Presents hexavalently the pseudo-tripeptide Lysψ(CH_2_N)-Pro-Arg coupled to a similar template as in HB-19: Ac-Lys-Ala-Lys-Pro-Gly-Lys-Ala-Lys-Pro-Gly-Lys-Ala-Lys-Pro-Gly-CONH_2_.

**Nucant 3: N3**

Presents pentavalently the pseudo-tripeptide Lysψ(CH_2_N)-Pro-Arg coupled to the polypeptide template containing Aib (2-aminoisobutyric acid): Ac-Lys-Aib-Gly-Lys-Aib-Gly-Lys-Aib-Gly-Lys-Aib-Gly-Lys-Aib-Gly-CONH_2_.

**Nucant 6: N6**

Presents hexavalently the pseudo-tripeptide Lysψ(CH_2_N)-Pro-Arg (a mixture in L and D configuration) coupled to the polypeptide template containing Aib: Ac-Lys-Aib-Gly-Lys-Aib-Gly-Lys-Aib-Gly-Lys-Aib-Gly-Lys-Aib-Gly-Lys-Aib-Gly-CONH_2_.

**Nucant 6L: N6L**

Presents hexavalently the pseudo-tripeptide Lysψ(CH_2_N)-Pro-Arg (all bonds in L position) coupled to a polypeptide template as in N6.

HB-19 and related Nucant pseudopeptides present pentavalently or hexavalently the pseudo-tripeptide Lysψ(CH_2_N)-Pro-Arg; ψ(CH_2_N) stands for a reduced peptide [9,16,43,44]. N6L has a similar template as N6 but the Lysψ-Pro units of the pseudo-tripeptide Lysψ(CH_2_N)-Pro-Arg moieties are all in L configuration, while in N6 the Lysψ-Pro units are a mixture in L and D configuration [44,49]. The nucleolin blocking affinity of constructs that present hexavalently the pseudo-tripeptide Lysψ(CH_2_N)-Pro-Arg (N6, N6L and N7) is 2-3 fold higher compared to the constructs that present pentavalently the same pseudo-tripeptide (HB-19 and N3).

### Analysis of nucleolin by immunoblotting

Nucleolin was analyzed by immunoblotting using either the monoclonal antibody (mAb) D3 against nucleolin (kindly provided by Dr. J. S. Deng) [6], or rabbit polyclonal antibodies directed against a synthetic peptide corresponding to the first 26 (MVKLAKAGKNQGDPKKMAPPPKEVEE) and the last 16 (GGGGDHKPQGKKTKFE) amino acid residues of human nucleolin as described before [8]. Nucleophosmin was revealed using rabbit monoclonal antibody EP1848Y (abcam). The murine mAb D3 and rabbit antibodies were revealed with horseradish peroxidase-conjugated sheep anti-mouse and goat anti-rabbit immunoglobulin (Jackson ImunoResearch), respectively. The reacting bands were visualized with an enhanced chemiluminescence (ECL) reagent and by exposure to autoradiography film (Amersham Biosciences). In some experiments, the presence of actin was monitored as a control with mAb anti-actin A-4700 (Sigma).

### Preparation of cytoplasmic and nuclear extracts

Cells washed in phosphate-buffered saline (PBS) were lysed in buffer E (20 mM Tris-HCl, pH 7.6, 150 mM NaCl, 5 mM MgCl_2_, 5 mM β-mercaptoethanol, protease inhibitor cocktail (Sigma) and 0.5% Triton X-100) and the nuclei were pelleted by centrifugation (1000 *g *for 5 min). For the preparation of nuclear extracts, the nuclear pellet was disrupted in buffer I (20 mM Tris-HCl, pH 7.6, 50 mM KCl, 400 mM NaCl, 1 mM EDTA, 5 mM β-mercaptoethanol, protease inhibitor cocktail, 1% Triton X-100, and 20% glycerol). Cytoplasmic and nuclear extracts were then centrifuged at 12,000 *g *for 10 min, and the supernatants (nucleoplasm) were stored at -20°C. Aliquots of crude cell extracts were diluted in 2 fold concentrated electrophoresis sample buffer containing SDS, and processed for immunoblotting analysis [9,10]. To monitor the profile of proteins in crude cells extracts, gels were stained with Brilliant Blue G-Colloidal Concentrate from Sigma [37].

### Gel-filtration chromatography

Three days after passage of HeLa P4 cells (10 × 150 cm^2 ^flasks), subconfluent cell monolayers were washed twice in PBS containing 1 mM EDTA before preparation of cytoplasmic extracts using 10 × 200 μl buffer E. For the gel filtration of surface nucleolin bound to the biotinylated HB-19 (HB-19/Btn), HeLa cells were first incubated with 5 μM of HB-19/Btn (45 min at room temperature) before preparation of extracts. Cytoplasmic extracts were diluted in PBS containing 1 mM EDTA then centrifuged at 12,000 *g *for 10 min, and filtered using Costar^® ^Spin-X^® ^centrifuge tubes filters (0.45 μm membrane pores size). Gel-filtration chromatography was carried out as previously described [50] on a GE Pharmacia fast protein liquid chromatography (FPLC) system. Briefly, a Superose™ 6 column (1.6 cm × 50 cm) was equilibrated in buffer GF containing 20 mM Tris/HCI, pH 7.6, 50 mM NaCl and 0.1% Triton X-100 at 0.5 ml/min. The column (bed volume 100 ml) was calibrated using cell extracts supplemented with gel filtration standard proteins from GE Healthcare Life Sciences: thyroglobulin (669-kDa), catalase (232-kDa) and BSA (67-kDa). Elution was in buffer GF with collection of 1-ml fractions/2 min, with a void volume (Vo) and total elution volume (Vc) at 30 ml and 114 ml, respectively. The void volume (Vo) was determined from the elution profile of Blue Dextran 2000 and total elution volume from the elution profile of Bromophenol blue. The sample (1 ml) was loaded on the column, and the eluate was monitored at 280 nm. Aliquots from each fraction were assayed for DPP IV activity by the cleavage of Gly-Pro-NH-Np (Sigma) in order to determine the peak of the 110-kDa DPP IV. The peaks of thyroglobulin, catalase and BSA were determined by polyacrylamide gel electrophoresis. The presence of nucleolin in various fractions was revealed by immunoblotting.

### Purification of the cell surface expressed 500-KDa complex containing surface nucleolin for microsequencing of nucleolin-associated proteins

Twenty-four hours after passage, CEM cells (10^9 ^cells) were washed extensively with PBS before incubation in culture medium (RPMI, 10% FCS) at room temperature for 30 min with 5 μM HB-19/Btn. After washing extensively in PBS containing 1 mM EDTA (PBS/EDTA), cytoplasmic extracts were prepared in lysis buffer E. The complex formed between cell-surface-expressed nucleolin and the HB-19/Btn was isolated by purification of extracts using avidin-agarose (Simon-Pure Immobilized Avidin from Pierce) in PBS/EDTA. After 2 h of incubation at 4°C, the samples were washed extensively with PBS/EDTA. The purified proteins were denatured by heating in the electrophoresis sample buffer containing SDS and analyzed by SDS-PAGE (7.5%). The proteins were transferred to a PVDF membrane before microsequencing the NH_2_-terminal ends (performed by the Protein-Sequencing Laboratory at Institut Pasteur, Paris).

### Triggering the production of pro-inflammatory cytokines by peripheral blood lymphocytes

Peripheral blood mononuclear cells (PBMC) from healthy donors were prepared by Ficoll-Hypaque density gradient centrifugation [45] and suspended in RPMI 1640 medium containing 1% (v/v) of human serum AB (from Invitrogen). PBMC (10^6 ^cells/0.5 ml) in the absence or presence of 10 μM of each of HB-19, N3, N6, and N7, or 1 μg/ml of dexamethasone (Sigma) were stimulated with 10^8 ^particles/ml of Heat-killed *Staphylococcus aureus *(HKSA; InvivoGen, San Diego, USA). The cultures were incubated at 37°C in a 5% CO_2 _incubator, and the level of TNF-α and IL-6 was monitored in the culture supernatants by ELISA (R & D Systems) at 20 hours post-stimulation.

### Inhibition of surface nucleolin function as a cell surface receptor for HIV-1 entry into HeLa CD4+ cells

Surface nucleolin serves as a low affinity receptor implicated in the binding and entry of HIV-1 particles into permissive cells. Consequently, HB-19 treatment of cells prevents HIV binding to cells and thus HIV entry and infection [9,16]. Here HIV-1 LAI entry was monitored in HeLa-CD4-LTR-LacZ cells containing the bacterial lacZ gene under the control of HIV-1 LTR. Virus entry and replication result in trans-activation of HIV-1 LTR by the viral Tat protein, leading to the expression of β-galactosidase. At 48 h post-infection, cell monolayers are lysed in a phosphate buffer containing Nonidet P-40 (Sigma) (1%; v ⁄ v) and assayed for β-galactosidase activity by measuring optical density at 570 nm [45].

### Analysis of the cell-surface-expressed nucleolin

Two days after seeding, subconfluent cells (about 5 × 10^6 ^cells/75 cm^2 ^flask) were incubated (45 min, 20°C) with 5 μM of HB-19/Btn. After washing extensively in PBS containing 1 mM EDTA (PBS-EDTA), nucleus-free cell extracts were prepared in lysis buffer E. The complex formed between cell-surface expressed nucleolin and HB-19/Btn was isolated by purification of the extracts using NeutrAvidin agarose (100 μl; Pierce Biotechnology) in PBS-EDTA. After 3 hours at 6°C, the avidin-agarose samples were washed extensively with PBS-EDTA. The purified surface nucleolin was eluted in the electrophoresis sample buffer containing SDS and analyzed by 10% SDS-polyacrylamide gel electrophoresis (SDS-PAGE). The presence of nucleolin was then revealed by immunoblotting using mAb D3 as described before [10,16]. All cells investigated in this study (MDA-MB-231, MDA-MB-435, LNCaP, HeLa, SW480, SW620, and T29) expressed the cell surface nucleolin assayed by this procedure.

### Immunofluorescence microscopy

Cells were plated 24 hours before the experiment in eight-well glass slides (Lab-Tek Brand; Nalge Nunc International, Naperville, IL). Cells were fixed with PFA/Triton X-100 solution (PFA/Triton) for staining intracellular biotinylated pseudopeptides (HB-19, N3, and N7) and nucleolar nucleolin [6,16]. Polylysine (0.01%) coated glass slides were used for adhesion of cells that proliferate in suspension. SW480 and SW620 cells plated in glass slides were cultured in the absence (control) or presence of 10 μM N6L for several days. Cells were either photographed as such or washed with PBS before fixation with PFA/Triton solution and processed for the detection of nuclear nucleolin using mAb D3. The secondary antibodies were the following: FITC-conjugated goat anti-mouse IgG (Sigma) and rabbit anti-biotin concentrate (IgG fraction; Enzo Dioagnostics, Inc., New York). The nuclei were stained with 4',6-diamidino-2-phenylindole (DAPI). It should be noted that artifactual results are observed when cells are fixed with methanol/acetate (3/1) to reveal intracellular localization of HB-19 or Nucant pseudopeptides, as it is the case for intracellular nucleolin [6,15].

### Analysis of DNA fragmentation in response to Nucant treatment

Extraction of nuclei in buffer I (prepared as above) from viable cells results in the recovery of chromatin-free nucleoplasm. However, during cell death when DNA fragmentation occurs, the low molecular weight DNA fragments are recovered in the nucleoplasm. Consequently, supernatant of nuclear extracts from cells undergoing programmed cell death contain low molecular weight DNA fragments, whereas the pellet of the nuclear extracts contain the high molecular weight chromatin. By this experimental approach, DNA fragmentation could be analyzed without interference of the bulky DNA [51]. For the preparation of the low molecular weight DNA fragments, nucleoplasm was incubated with 1 mg/ml RNase for 1 hour at 50°C, then 0.5 mg/ml proteinase K for 1 hour at 50°C, followed by extraction with phenol/chloroform/isoamyl alcohol (25:24:1) and precipitation with ethanol. The DNA pellet was resuspended in electrophoresis buffer (10 mM Tris-HCI, pH 8.0, 1 mM EDTA) and analyzed by electrophoresis on 1.5% agarose gels containing 0.5 pg/ml ethydium bromide.

### mRNA expression monitored by reverse transcription-polymerase chain reaction (RT-PCR)

SW620 cells were cultured in the absence or presence of N7 before extraction for total RNA using RNeasy Mini Kit (Qiagen) according to the manufacturer's instructions. RT was carried out with oligo(dT) and 2-4 μg of total RNA using Superscript II Reverse Transcriptase (Invitrogen). PCR was performed in a RoboCycler 96 (Stratagene, La Jolla, CA, USA) with specific primers for human nucleolin (referred to as NCL) 5'-TTGAATTCATCATGGTGAAGCTCGCGAAGGC-3' and 5'-TAGGGCCCAGGCTCTTCCTCCTC-3' (835 bp); glyceraldehyde-3-phosphate dehydrogenase (GAPDH) 5'-TGAAGG-TCGGAGTCAACGGATTTGGT-3' and 5'-CATGTGGGCCATGAGGTCCA-CCAC-3' (983 bp); nucleophosmin (NPM or B23) 5'-TGGTTCTCTTCCCAAAGTGG-3' and 5'-TAAAACCAAGCAAAGGGTGG-3' (320 bp); matrix metalloproteinase-2 (MMP-2) 5'-GTGCTGAAGGACACACTAAAGAAGA-3' and 5'-TTGCCATCCTTCTCAAAGTTGTAGG-3' (580 bp); matrix metalloproteinase-9 (MMP-9) 5'-CACTGTCCACCCC TCAGAGC-3' and 5'-GCCACTTGTCGGCGATAAGG-3' (243 bp); tissue inhibitor of metalloproteinase 1 (TIMP-1) 5'-ATCCTGTTGTTGCTGTGGCTGATAG-3' and 5'-TGCTGGGTGGTAACTCTTTATTTCA-3' (667 bp); tissue inhibitor of metalloproteinase 2 (TIMP-2) 5'-AAACGACATTTATGGCAACCCTATC-3' and 5'-ACAGGAGCCGTCACTTCTCTTGATG-3' (405 bp). PCR amplification conditions were: 95°C for 5 min, 30 cycles at 95°C for 30 sec, 60°C for 30 sec and 72°C for 45 sec, and 72°C for 5 min (for NCL and GAPDH), 95°C for 5 min, 30 cycles at 95°C for 30 sec, 53°C for 30 sec and 72°C for 30 sec, and 72°C for 5 min (for NPM); 95°C for 5 min, 35 cycles at 95°C for 45 sec, 56°C for 45 sec and 72°C for 45 sec, and 72°C for 5 min (for MMP2 and MMP9); 95°C for 5 min, 30 cycles at 95°C for 45 sec, 59°C for 1 min and 72°C for 1 min 30 sec, and 72°C for 5 min (for TIMP1 and TIMP2).

### Statistical analysis

The significance of variability between the results of each group and its corresponding control was determined by unpaired t-test and Mann-Witney Anova. All results are expressed as mean ± standard errors of the means from at least two independent experiments.

## Results and Discussion

### The cell-surface expressed nucleolin exists in a complex of high molecular weight

**Figure 1:**
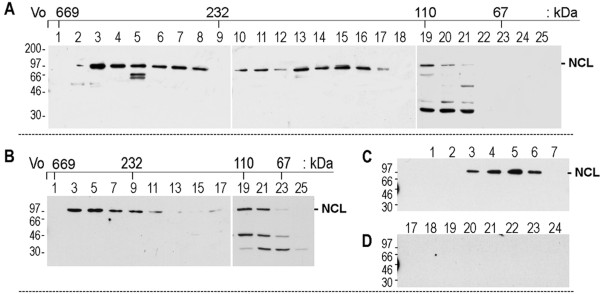
**Gel filtration of HeLa cell extract containing cytoplasmic and plasma membrane proteins**. A. The preparation of Triton-X100 soluble cell extract was analyzed by gel filtration using a superose-6 column. One ml fractions (numbered from 1 to 25) were collected after the elution of the void volume (Vo), and the presence of nucleolin was monitored by immunoblotting using rabbit polyclonal antibodies against the NH_2_-terminal peptide of nucleolin. The numbers 232, 110 and 67 on the top indicate the position of protein markers of molecular weight 669-, 232-, 110-, and 67-kDa. NCL on the right shows the main nucleolin band at position 100-kDa. The other low molecular weight bands represent the cleaved products of nucleolin. B. HeLa cells were first incubated with 5 μM of HB-19/Btn at room temperature before preparation of cell extracts. Gel filtration was carried out as in section 1A, but only one every other sample was analyzed by immunoblotting. C/D. The peak of nucleolin eluting at an apparent molecular weight of 500-kDa is complexed with HB-19/Btn. Fractions 1-7 and 17-24 from the gel filtration experiment described in section B were purified by affinity chromatography using Avidin agarose and analyzed by immunoblotting. On the right of the different gels is the position of the molecular weight markers. All experimental procedures were as described in Methods.

The elution profile of nucleolin was investigated by gel filtration of Triton-X100 soluble cell extracts of cytoplasmic and plasma membrane proteins. Immunoblotting aliquots from various fractions then revealed the presence of three nucleolin peaks located within fractions 3-8, 10-17 and 19-21, which correspond to the elution profile of proteins of apparent molecular weights of 500-, 200-, and 100-kDa, respectively (Figure 1A). The 100-kDa nucleolin peak is in accord with its molecular mass after SDS-PAGE analysis, and should correspond to the monomeric form of nucleolin. On the other hand, the higher molecular weight peaks of nucleolin could correspond to nucleolin multimers or nucleolin complexed with other proteins.

In order to differentiate between nucleolin expressed on the cell surface from that found in the cytoplasm, we carried out a similar gel filtration experiment but using extracts from cells that were preincubated at room temperature with biotinylated HB-19 (HB-19/Btn). At reduced temperatures, HB-19 binds surface nucleolin and forms a stable complex but it is not internalized, thus allow the differentiation between surface and cytoplasmic nucleolin. After gel filtration, nucleolin was recovered in two main peaks located in fractions 3-9 and 19-23 corresponding to apparent molecular weight of 500- and 100-kDa, respectively, whereas the peak at the molecular weight of 200-kDa was greatly reduced (Figure 1B). For the recovery of nucleolin complexed to HB-19/Btn, fractions 1-7 and 17-24 were purified by affinity chromatography using avidin-agarose Figure 1C and 1D). The results indicate that the peak of nucleolin eluting at an apparent molecular weight of 500-kDa represents surface nucleolin, whereas the 100-kDa peak corresponds to nucleolin present in the cytosol. The marked reduction of the 200-kDa nucleolin peak might reflect the shift of this form of nucleolin towards the cell surface during the incubation period of cells with HB-19/Btn. Taken together, these results indicate that the existence of surface nucleolin in a high molecular weight complex is independent of ligand binding to surface nucleolin.

### Identification of proteins associated with the surface expressed nucleolin in the 500-kDa complex

The 500-kDa complex containing surface nucleolin was purified from the cell surface by the capacity of HB-19/Btn to bind specifically surface nucleolin [9,16,52]. This 500-kDa complex is highly stable but it becomes dissociated in the presence of the ionic detergent SDS. Consistent with our previous results, we first confirmed that nucleolin is the only HB-19 binding protein band by ligand blotting using HB-19/Btn [9,16,52]. Secondly, by staining of the SDS-PAGE gel we revealed the presence of several other protein bands, which were processed for NH_2_-terminal microsequencing (Table 2 Table 3; Additional file 2, Figure S2). Such surface nucleolin associated proteins were identified as two Wnt related proteins, the 80-kDa subunit of the Ku autoantigen, the signal recognition particle subunits SRP72 and SRP68, a protein described in the literature as p32 or the hyaluronan binding protein 1 (HABP1) or as the receptor for complement component C1q (gC1q-R), and ribosomal proteins S4 and S6. Like surface nucleolin, gC1q-R is glycosylated and lacks GPI anchor or a transmembrane segment. The mature gC1q-R protein localized at the cell surface lacks its first 73 amino acid residues [53]. Consistent with this, the NH_2_-terminal sequence that we obtained for gC1q-R purified with the cell surface nucleolin starts at amino acid residue 74 (Table 2). The most abundant location of p32/gC1q-R is in the mitochondria where it is shown to be a critical regulator of tumor metabolism via maintenance of oxidative phosphorylation [54]. However, the effect of surface gC1q-R on its mitochondrial counterpart is not yet elucidated. Cell surface expressed gC1q-R can serve as a receptor for diverse proinflammatory ligands, and as a binding protein for a number of functional antigens of viral and bacterial origin. Particularly, *Staphylococcus aureus *via its protein A uses gC1q-R as a cellular protein for attachment and/or entry into host cells [55]. Previously, the urokinase receptor and caseine kinase have been reported to colocalize with surface nucleolin [56]. However, we did not identify them among the other nucleolin-associated proteins recovered from the surface of cells (Table 2). This latter could be the consequence of our failure to microsequence some of the protein bands because of poor recovery from the PVDF membrane or blockade of their NH_2_-terminal end (see the Additional file 2, Figure S2). On the other hand, we cannot exclude the possibility that nucleolin in the 200-kDa peak is complexed with urokinase, which like HB-19 binds the RGG domain of nucleolin [20].

**Table 2:** Identification of nucleolin associated proteins in the 500-kDa complex.

**Protein Band (MW)**	**Obtained Sequence**^**1**^**/Medline Sequence**^**2**^
Band 5 (90-kDa)	MHRPFXX^1^
	1-MHRNFRK^2 ^= Wnt related protein A^3^
	
Band 6 (85-kDa)	MRPMTQIIVQD^1^
	1-MRPMTFIVGLK^2 ^= Wnt related protein B^4^
	
Band 7 (80-kDa)	VRSGNKAAVVLXMDVXFTMS^1^
	1-MVRSGNKAAVVLCMDVGFTMS = Ku80^5^
	
Band 8 (72-kDa)	ASGGSGGVXVPA^1^
	1-MASGGSGGVSVPA^2 ^= SRP72^6^
	
Band 9 (68-kDa)	AAEKQVPGGXXGGGS^1^
	1-MAAEKQVPGGGGGGGS^2 ^= SRP68^6^
	
Band 11 (32-kDa)	LHTDGDKAFVDFLSD^1^
	74-LHTDGDKAFVDFLSD^2 ^= p32/p33/HABP1/gC1q-R^7^
	
Bands 12 (25-kDa):	ARGPKKHLK^1^
	1-MARGPKKHLK^2 ^= S4^8^
	
	KLNISFPAL^1^
	1-MKLNISFPAL^2 ^= S6^8^

**Figure 2:**
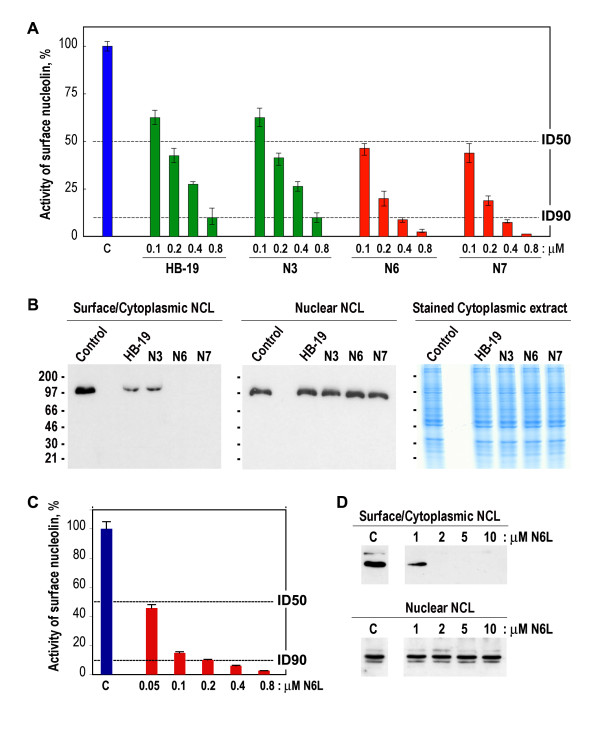
**HB-19 and related Nucant pseudopeptides antagonize surface nucleolin and trigger its down regulation**. A. Multivalent pseudopeptides block the biological function of surface nucleolin for HIV entry. HeLa CD4+ cells in the absence (histogram C) or presence of 0.1, 0.2, 0.4 and 0.8 μM of HB-19, N3, N6, and N7 were assayed for HIV-1 entry into cells. At 48 h post-infection, the β-galactosidase activity was measured in cell extracts directly to monitor HIV entry (Methods). The abscissa gives % Activity of surface nucleolin calculated from the optical density measurements at 570 nm. The mean standard deviation (± SD) of triplicate samples is shown. B. Down regulation of surface nucleolin by multivalent pseudopeptides. Two days after passaging MDA-MB 231 cells were untreated (lanes Control) or treated with 10 μM of HB-19, N3, N6, and N7 for 16 hours. Cytoplasmic (containing cytoplasmic as well as surface nucleolin) and nuclear extracts were analyzed by immunoblotting using mAb D3 as described (Methods)(panels Surface/cytoplasmic NCL, Nuclear NCL). The corresponding cytoplasmic extracts from section B were stained with Brilliant Blue G-Colloidal Concentrate staining (panel Stained Cytoplasmic extract). C. Hexavalent N6L pseudopeptide blocks the biological function of surface nucleolin for HIV entry in a dose dependent manner. HeLa CD4+ cells in the absence (histogram C) or presence of 0.05, 0.1, 0.2, 0.4 and 0.8 μM of N6L were assayed for HIV-1 entry into cells as in section A. D. Down regulation of surface nucleolin by N6L in HeLa CD4+ cells in a dose dependent manner. HeLa cells in the absence (histogram C) or presence of 1, 2, 5, and 10 μM of N6L were cultured for 7 hours before preparation of cell extracts and immunoblotting as in section B. The intensity of nucleolin protein bands was quantified by using the NIH image software. The values for the statistical significance in section A and C are the following: p is < 0.1 for HB-19 and N3 at 0.1 μM, whereas p is < 0.001 at higher concentration of HB-19 and N3; p is < 0.001 for N6, N7, and N6L at the various concentrations used for the assay.

By the capacity of the HB-19/Btn to bind surface nucleolin, the 500-kDa complex was recovered from the cell surface and purified using avidin-agarose. After analysis by SDS-PAGE, several protein bands of 90-, 85-, 80-, 72-, 68-, 32, and 25-kDa were processed for NH_2_-terminal microsequencing (Methods; Additioal file 2, Figure 2S). These protein bands were identified as Wnt related proteins, Ku80, SRP68, SRP72, p32/p33/HABP1/gC1q-R, and ribosomal proteins S4 and S6.

^1 ^The obtained NH_2_-terminal sequence.

^2 ^The NH_2_-terminal sequence in the Medline.

^3 ^The obtained MHRPFXX sequence shares homology to the N-terminal sequence of human Wnt-7b (MHRNFR) [87].

^4 ^The obtained MRPMTQIIVQD sequence is homologous in its first 5 amino acid residues with the N-terminal sequence (MRPMTFIVGLK) of Wnt-1 of the Mexican axolotls Ambystoma mexicanum [88]. Consequently, the isolated protein appears to correspond to a human Wnt-related protein that remains to be described.

^5 ^The 80-kDa subunit of the Ku autoantigen [89]

^6 ^The 68- and 72-kDa subunit of the signal recognition particle [60].

^7 ^Referred in the literature as the protein p32 associated with the splicing factor SF2, the hyaluronan binding protein 1 (HABP1) and the receptor for complement component C1q (gC1q-R)[90].

^8 ^Ribosomal proteins S4 and S6 [91].

**Table 3:** Characteristics of proteins associated with the cell surface expressed nucleolin.

**Wnt related protein A and B**. Protein A shares homology with human Wnt-7b [87], while protein B shares homology with Wnt-1 of the Mexican axolotl Ambystoma mexicanum [88]. The Wnt proteins are a family of secretory glycoproteins mostly associated with cell membranes and the extracellular matrix. They are implicated in proliferation and differentiation of both normal and malignant cells. Many members of the WNT gene family, including WNT-7, are up-regulated in bladder and breast carcinoma and as well as in chronic lymphocytic leukemia, suggesting involvement of Wnt signaling pathways in tumorigenesis [92-94].
**80-kDa subunit of Ku**. The Ku autoantigen is a heterodimeric protein made up of 70- and 80-kDa subunits. Besides its central importance to DNA repair, Ku has a key role in a number of other fundamental cellular processes such as telomere maintenance, transcription and cell death [89]. The cell surface expressed Ku80 is detected in a variety of tumor cells, including leukemia and solid tumor cells [95]. Surface Ku contributes to adhesion and invasion of tumor cells thus potentiating tumor metastasis [59].

**SRP68 and SRP72**. These are the 68- and 72-kDa subunit in the signal recognition particle, a ribonucleoprotein complex composed of 7S RNA and 6 proteins of 9-, 14-, 19-, 54-, 68-, 72-kDa. SRP comprise the major cellular machinery that mediates the cotranslational targeting of proteins to cellular membranes [60,61]. SRP68 and SRP72 are functionally linked. Experimental evidence has demonstrated that SRP68 binds SRP72 and forms a highly stable heterodimer [96].

**32-kDa protein referred to as p32/p33, HABP1, or gC1q-R**. A multifunctional and muticompartmental cellular protein that was originally isolated based on its copurifiation with the nuclear splicing factor SF2 (p32/p33). It has also been described on the cell surface as the hyaluronan binding protein 1 (HABP1) and the receptor for complement component C1q (gC1q-R) [97]. Hyaluronan is a glycosaminoglycan that with its surface receptors regulate tumor cell adhesion, migration and invasion [98]. Preferentially over expressed in adenocarcinoma cells, gC1q-R is a molecular target in tumor cells and tumor stroma [90,99]. In addition to cancer, gC1q-R is considered to play an important role in bacterial infections and inflammation [54,55].

**Ribosomal proteins S4 and S6**. S4 and S6 are respectively components of the 40S and 60S ribosomal subunits, which generate ribosomal 80S subunit implicated in the cellular process of translation. Ribosomal proteins with nucleolin are also implicated in the processing and assembly of pre-ribosomal particles in the nucleolus. Like HB-19, several ribosomal proteins bind nucleolin via its RGG domain [9,91]. The role, if any, of the surface expressed S4 and S6 remains to be investigated. It is worthwhile to note that several ribosomal protein genes have been reported to act as cancer genes in zebrafish [100].

Interestingly, most of surface-nucleolin associated proteins are implicated in cell signaling, tumor cell adhesion, migration, invasion, cell death, and inflammation (Table 3). In addition, Ku and nucleolin are important autoantigens in patients with systemic lupus erythematosus and other systemic autoimmune disorders [57,58], thus suggesting the potential implication of the 500-kDa complex in autoimmune diseases. The mechanism by which Ku is translocated to the cell surface is not known. However, it is of interest to note that in the cytoplasm both Ku and nucleolin are packaged within small vesicles, and are translocated to the cell surface by a mechanism independent of the conventional endoplasmic reticulum/Golgi secretory pathway [6,59]. In view of the observation that SRP68 and SRP72 are associated with the high molecular weight nucleolin complex (Table 2), and the implication of SRP in the cotranslational delivery of nascent secretory and membrane proteins [60,61], it is tempting to speculate that SRP could coordinate active translocation of Ku and nucleolin towards the plasma membrane.

None of the isolated proteins in the 500-kDa complex including nucleolin possess a hydrophobic transmembrane domain to account for anchorage in the plasma membrane. In spite of this, the 500-kDa complex is tightly associated with the plasma membrane because extensive washing of cells with high concentrations of EDTA, EGTA, or NaCl have no effect. On the other hand, the 500-kDa complex is readily recovered by solubilization of the plasma membrane with non-ionic detergents, Triton X-100 or NP-40, thus indicating that its association with the cell surface is through non-covalent interactions. The surface nucleolin in the 500-kDa complex should be associated, directly or indirectly, to an integral membrane protein partner that holds this complex to coordinate clustering of surface nucleolin along with active endocytosis of various nucleolin-binding ligands via lipid rafts [10,17,19]. A potential candidate for a transmembrane protein partner of the 500-kDa complex is the low-density lipoprotein (LDL) receptor related protein (LRP1), which is a large scavenger receptor mediating endocytosis of various biological components and is largely implicated in cytoskeleton organization [7,62,63]. The link between the 500-kDa complex and LRP1 could be apoplipoprotein E-enriched LDL that in addition to LRP1 binds also surface expressed nucleolin. Accordingly, anti-nucleolin antibody has the capacity to inhibit significantly the binding of LDL to the cell surface [64]. Interestingly, active internalization of specific surface nucleolin ligands (midkine, pleiotrophin, lactoferrin, HB-19) is dependent on the expression of LRP-1 [7,15,17-19,21,65]. Moreover, the expression of surface nucleolin appears to be dependent on the expression of LRP1. For example, in Chinese hamster ovary CHO LRP1-null cells, although nucleolin is present abundantly in the nucleus and in the cytoplasm, it remains undetectable at the cell surface (Additional file 3, Figure S3). Consequently, in the absence of surface nucleolin in such LRP1-null cells, ligands of nucleolin become internalized by a receptor-independent passive process (Additional file 3, Figure S4). These reports and observations suggest that LRP1 might be one of the potential transmembrane anchored partners that allow surface expression of nucleolin in the 500-kDa complex. The mechanism by which LRP1 expression could coordinate the expression of nucleolin at the cell surface remains to be elucidated.

### HB-19 and related Nucant pseudopeptides exert distinct inhibitory effects on different types of tumor cells in culture

HB-19 presents pentavalently the pseudo-tripeptide Lysψ(CH_2_N)-Pro-Arg coupled to a peptide template. In order to improve the biological activity of HB-19, several new constructs named Nucant (for nucleolin antagonist) were generated (Methods). These constructs present either pentavalently (N3) or hexavalently (N6, N6L and N7) a similar pseudo-tripeptide Lysψ(CH_2_N)-Pro-Arg as HB-19 (Table 1; Additional file 1, Figure S1). The nucleolin-antagonist effect of HB-19 and Nucant constructs were assayed conveniently by their capacity to block HIV-1 entry into permissive cells [10,16,45]. Accordingly, we show that HB-19 and related Nucant constructs inhibit HIV-1 entry in a dose dependent manner (Figure 2A and 2C). The inhibitory activity of N3 is similar to that of HB-19. On the other hand, the inhibitory activity of N6/N7 and N6L is at least 2- and 4-fold more active, respectively, compared to the pentavalent constructs N3 and HB-19. After binding surface nucleolin, Nucant constructs like HB-19 [37] are internalized by an active process and accumulate in the cytoplasm without entering the cell nucleus. An example is presented in the Additional file 4, Figure S5 showing the cytoplasmic entry of the biotinylated HB-19, N3, and N7 in MDA-MB 231.

Finally, treatment of cells with HB-19 and Nucant constructs results in a drastic down regulation of surface/cytoplasmic nucleolin without affecting nuclear nucleolin (Figure 2B). Interestingly, the reduction of surface/cytoplasmic nucleolin is greater in cells treated with N6 and N7 compared to cells treated with HB-19 and N3. In fact, the level of surface/cytoplasmic nucleolin is almost completely abolished in cells treated with either N6 or N7. This is due to a selective reduction of surface/cytoplasmic nucleolin, since the profile of cytoplasmic proteins assayed by Brilliant Blue G-Colloidal Concentrate staining is comparable in the untreated control and HB-19 or Nucant treated cells (Figure 2B). Consistent with its higher activity as an antagonist of surface nucleolin (Figure 2C), N6L treatment causes a drastic down regulation of surface but not nuclear nucleolin in a dose dependent manner (Figure 2D). At 1 μM of N6L surface nucleolin level is reduced by more than 80%, whereas at 2 μM concentration surface nucleolin is no longer detectable. Taken together, these results indicate that selective reduction of surface/cytoplasmic nucleolin occurs independently of nuclear nucleolin, which further illustrates that HB-19 and Nucant pseudopeptides exert their inhibitory effects without toxicity. We have recently reported that in spite of reduction of surface/cytoplasmic nucleolin protein, nucleolin mRNA is continuously induced but it is not translated [15]. The molecular mechanism of such a specific translational block on nucleolin mRNA in HB-19 and Nucant treated cells remains to be investigated. However, it is tempting to speculate that nucleolin mRNA might require the nucleolin protein for its translation as it is the case for metalloproteinase-9 (MMP-9) and *bcl2 *oncogene mRNA [4,5].

During our initial studies to test the inhibitory activity of HB-19 and related Nucant pseudopeptides on various tumor cell lines, we noticed that they exert distinct inhibitory mechanisms depending on the malignant tumor cell type. Indeed, in epithelial tumor cells they induce reversion of the malignant phenotype while in leukemia cells they trigger a programmed cell death. Table 4 gives a summary of the inhibitory activity of hexavalent Nucant consructs N6/N7 compared to N6L on various epithelial tumor cell lines and on leukemia cells. Such Nucant constructs inhibited markedly the growth of all types of tumor cell lines after 3 days of treatment, but more strikingly they selectively induced cell death only in leukemia cells after 24 hours of treatment. In the group of the epithelial cell lines, we noticed that Nucant treatment could affect adhesion of cells (MDA-MB 435, MDA-MB 231, LNCaP, and HeLa cells), restore contact inhibiton (TIII, MDA-MB 435, and MDA-MB 231 cells), inhibe spreading (SW480 and SW620 cells) and migration (MDA-MB 435, MD1-MB 231) of tumor cells. Consequently, we used these different cell lines to demonstrate the distinct inhibitory action of HB-19 and related Nucant pseudopeptides.

**Table 4:** Inhibitory activity of hexavalent Nucant pseudopeptides N6, N7, and N6L in tumor cell lines of different origins.

Cell line	Tumor cell origin	% Growth inhibition	% Cell death
		After 3 days (N6/N7 - N6L)	After 24 hours (N6/N7 - N6L)
MDA-MB 231	Hu breast cancer	92 - 95%	< 5%
MDA-MB 435	Hu breast cancer	58 - 66%	< 5%
LNCaP	Hu prostate cancer	88 - 90%	< 5%
HeLa	Hu cervical cancer	46 - 60%	< 5%
SW480	Hu colon carcinoma	25 - 35%	< 5%
SW620	Hu colon carcinoma	45 - 55%	< 5%
TIII	Mu melanoma cells	55 - 83%	< 5%
			
HuT 78	Hu cutaneous T cell leukemia	62 - 83%	44 - 55%
Jurkat	Hu T-cell leukemia	80 - 85%	45 - 60%
RAJI	Hu Burkitt lymphoma	75 - 95%	42 - 71%
HL60	Hu acute promyelocytic leukemia	65 - 80%	35 - 48%
T29	Mu T-cell lymphoma	85 - 95%	45 - 65%

Epithelial (MDA-MB 231, MDA-MB 435, LNCaP, HeLa, SW480, SW620, TIII) and leukemia (HuT 78, Jurkat, RAJI, HL60, and T29) cell lines were cultured in the absence or presence of N6 or N7 (20 μM) or N6L (10 μM) to test their inhibitory activity on cell growth (by measuring viable cell number after 3 days of culture) and cell death (by monitoring the trypan blue uptake after 24 hours of treatment of subconfluent epithelial and freshly passaged leukemia cells). The number of viable cells in untreated control samples was used to calculate the % inhibition of cell growth and % cell death. Hu and Mu stand for human and murine origin, respectively. The mean percentage values of at least 2 independent experiments are presented.

### The inhibitory action of HB-19 and related Nucant pseudopeptides on the production of pro-inflammatory cytokines by human blood lymphocytes in response to stimulation by heat inactivated *Staphylococcus aureus*

**Figure 3:**
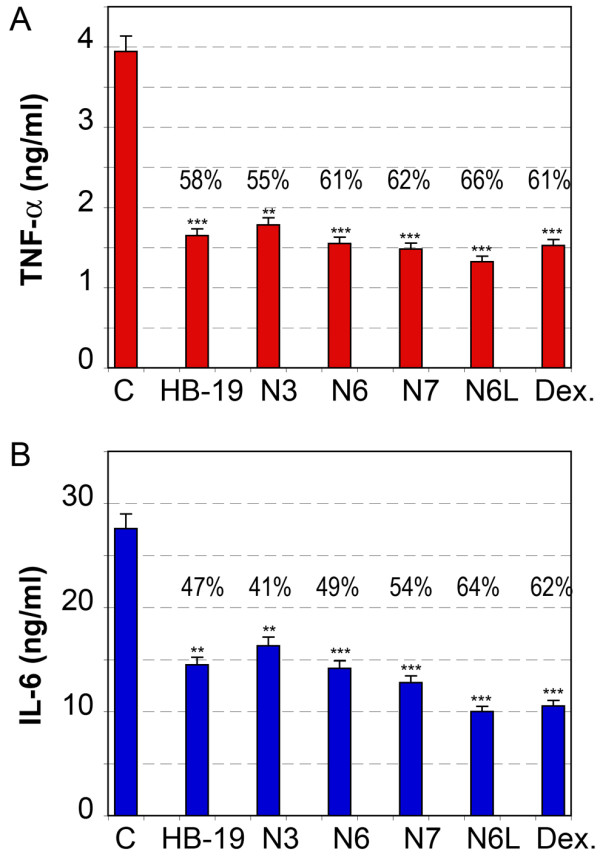
**HB-19 and related Nucant pseudopeptides inhibit the production of TNF-α and IL-6 by peripheral blood lymphocytes**. The production of pro-inflammatory cytokines in PBMC cultures stimulated with HKSA was carried out in triplicate in the absence (Control) or presence of 10 μM of each of HB-19, N3, N6, N7, N6L, or 1 μg/ml of dexamethasone (Dex.)(Methods). The level of TNF-α (section A) and IL-6 (section B) was monitored in culture supernatants by ELISA. The data presents the mean ± SD of triplicate samples. The degree of inhibition is indicated at the top of each histogram. Statistical significance: **p < 0.01, ***p < 0.001.

Surface nucleolin does not dissociate from the other proteins in the 500-kDa complex in the presence of non-ionic detergents thus indicating that these proteins are bound together. This and the capacity of HB-19 to bind surface nucleolin without dissociating the 500-kDa complex suggest that the binding of HB-19 to surface nucleolin could change the organization of the 500-kDa complex, and thus interfere with the proper functioning of nucleolin and the nucleolin associated proteins. In order to test this hypothesis, we investigated the capacity of the nucleolin antagonist pseudopeptides to interfere with one of the functions of gC1q-R as a cellular receptor of *Staphylococcus aureus *[55]. Human peripheral blood lymphocytes in the absence presence of nucleolin antagonist pseudopeptides were stimulated by Heat-killed *Staphylococcus aureus *(HKSA) and the production of proinflammatory cytokines TNF-α and IL-6 was monitored in culture supernatants at 20 hours post-stimulation (Figure 3). As a control for the inhibition of the inflammatory response, lymphocytes were treated with dexamethasone, which is a glucocorticoid drug with a known potent anti-inflammatory and immunosuppressant activity. We show that nucleolin antagonist pseudopeptides inhibit production of TNF-α and IL-6 in HKSA-stimulated primary lymphocytes by 55-66% and 41-64%, respectively, while dexamethasone inhibits by 61 and 62%, respectively (Figure 3). A similar inhibitory effect is observed when cells are first treated with HB-19 and Nucant pseudopeptides at 20°C, washed to remove unbound pseudopeptides before addition of HKSA at 37°C (data not shown). Therefore, by targeting surface nucleolin it is possible to interfere indirectly with the functioning of the other proteins associated with nucleolin in the 500-kDa complex, which is logical when considering the various inhibitory mechanisms triggered by HB-19 and Nucant pseudopeptides. Our results suggest that the inhibitory action of these pseudopeptides is mediated by their capacity to interact with surface expressed nucleolin rather than with HKSA. Nevertheless, we cannot exclude the possibility that the interaction of these pseudopeptides with nucleolin could trigger some downstream signaling event that inhibits production of TNF-α and IL-6.

The inhibitory activity on the production of inflammatory cytokines provide an important contribution to the overall anti-tumorigenic action of HB-19 and related Nucant pseudopeptides, since inflammation could constitute a risk factor for a variety of epithelial cancers by generation of free radicals, stimulation of cytokines, chemokines, and growth and angiogenic factors [66]. It is of interest to note that surface nucleolin is an eukaryotic receptor for the adhesin intimin-gamma of enterohemorrhagic *Escherichia coli *[11,67]. In view of this and the capacity of nucleolin antagonist pseudopeptides to block functioning of gC1q-R, we suggest that HB-19 and related Nucant pseudopeptides could also provide efficient inhibitors of pathogenic bacteria.

### Nucant pseudopeptides inhibit cell adhesion and spreading of human carcinoma cells

**Figure 4:**
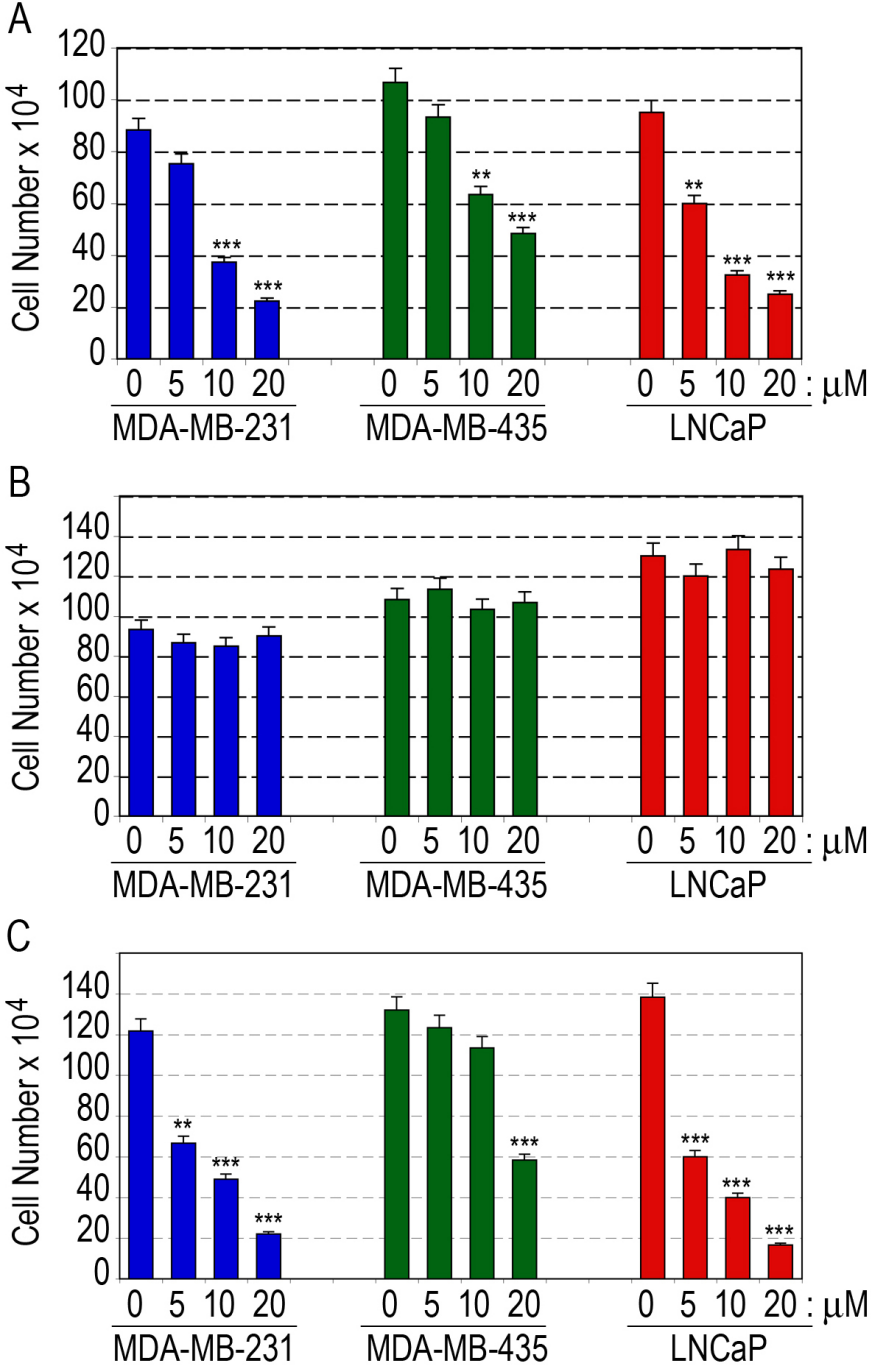
**The inhibitory effect of Nucant on cell adhesion occurs without any apparent cytotoxicity**. A. Inhibition of Cell adhesion by N6. MDA-MB 231, MDA-MB-435 and LNCaP (1 × 10^6^) cells were passaged in 25 cm^3 ^flasks in the absence (lanes 0 μM for control) or presence of 5, 10, and 20 μM of N6. After 16 hours of culture, adherent cells were trypsinized and the number of cells in each flask was monitored in the presence of trypan blue. B. Cell multiplication is not affected in Nucant cultured cells. Cells were seeded at 2 × 10^5 ^cells in 25 cm^3 ^flasks. When cells reached subconfluency (after 48 hours), they were further cultured for 24 hours in the presence of different concentrations of N6 before counting. C. The effect of Nucant treatment on cell proliferation. Seven hours after seeding (1 × 10^5 ^cells in 25 cm^3 ^flasks), cells were cultured in the presence of different concentrations of N6 and counted after four days. The ordinates give the mean ± SD of triplicate samples. Statistical significance: **p < 0.01, ***p < 0.001.

At concentrations that Nucant constructs do not affect the viability of several epithelial carcinoma cells, they inhibit attachment of cells to the plastic culture flask. A typical experiment is presented in Figure 4A showing dose dependent inhibitory effect of N6 on the attachment of human breast (MDA-MB-231 and MDA-MB-435) and prostate (LNCaP) carcinoma cells. At 10 μM of N6, the degree of adhesion inhibition in MDA-MB-231, MDA-MB-435 and LNCaP cells is 58, 41, and 66%, respectively. As a consequence of cell adhesion, culturing of cells with Nucant results in a dose dependent significant inhibition of cell growth, and moreover could restore contact inhibition (Figure 4C; Additional file 5, Figure S6). Similarly, Inhibition of cell proliferation of melanoma TIII cells by Nucant pseudopeptides is highly correlated with their capacity to inhibit cell adhesion. Consistent with the capacity to block the biological function of surface nucleolin at lower concentrations (Figure 2), the inhibitory effects were more pronounced with N6L compared to N6 (Table 5). It should be noted that HB-19 and related Nucant pseudopeptides are devoid of any significant cytotoxicity against such epithelial tumor cell lines in culture. For an example, the viable cell number is not affected when subconfluent MDA-MB-231, MDA-MB-435 or LNCaP cell monolayers are cultured for 24 hours in the presence of different concentrations of N6 (Figure 4B). Our results suggest that inhibition of cell adhesion is one of the inhibitory mechanisms of action of HB-19 and Nucant pseudopeptides leading to inhibition of cell proliferation of epithelial-like tumor cells. It is of interest to note that adhesion of tumor cells on the vasculature of endothelium is a critical and a decisive step in tumor cell invasion and metastasis. In this respect, Figure S7 in the Additional file 5 presents a typical experiment showing that Nucant treatment could down regulate motility of tumor cells as analyzed by a wound healing scratch assay [68]. Finally, adhesion of tumor cells to platelets also leads to the formation of micro-thrombin, which facilitates the metastasis process by allowing tumor cells to arrest in blood stream and to adhere to vascular wall [69,70].

**Table 5:** Inhibitory activity of N3, N6, and N6L on melanoma TIII cell adhesion and proliferation.

Nucant	% Inhibition of cell	
	
	Adhesion	Proliferation
N3: 5 μM	No effect	No effect
N3: 10 μM	15%	10%
N3: 20 μM	24%	36%
		
N6: 2.5 μM	15%	12%
N6: 5 μM	26%	20%
N6: 10 μM	38%	32%
N6: 20 μM	55%	45%
		
N6L: 2.5 μM	39%	45%
N6L: 5 μM	49%	56%
N6L: 10 μM	65%	80%
N6L: 20 μM	72%	85%

Epithelial melanoma TIII cells were cultured in the absence or presence of different concentrations of N3, N6, and N6L to monitor inhibition of cell adhesion (after 5 hours of cell seeding) and cell proliferation (after 3 days of culture). The number of viable cells in untreated control samples was used to calculate % inhibition of cell adhesion and proliferation. The mean percentage values of at least 2 independent experiments are presented.

### The inhibitory effect of Nucant on the spreading of human colon carcinoma cells is associated with down regulation of MMP-9 and nucleolin transcripts

**Figure 5:**
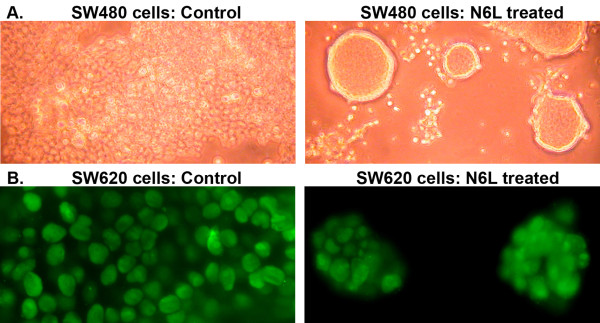
**N6L treatment results in a marked phenotypic change in human colon carcinoma cells giving typical circular colonies instead of spreading and growing in several layers**. SW420 (in section A; phase contrast images) and SW620 (in section B, immunofluorescence images) cells were cultured in the presence of 10 μM N6L for 3 days before analysis (Methods). The immunofluorescence images show nucleolin in the nuclei of cells revealed with mAb D3. The images were taken at 100- and 1000-fold magnification in section A and B, respectively.

In these experiments we used SW480 and SW620 cells that are established from a primary and a metastatic colon carcinoma, respectively. These cells were studied in this section because of a particular effect of pseudopeptides on the spreading of such tumor cells. Both cell types could spread on the plastic but piled up in cell culture forming clusters of several layers of cells. Treatment of both SW480 and SW620 cells with N6L and N7, that present hexavalently the pseudo-tripeptide Lysψ(CH_2_N)-Pro-Arg, resulted in growth inhibition of cells (Table 6). In addition, Nucant treatment resulted in a marked inhibitory effect on spreading SW480 and SW620 cells which formed isolated circular colonies (Figure 5). The inhibitory effect of Nucants on cell proliferation and spreading was consistently observed in SW480 and SW620 cells. In general, SW620 cells appeared to be more sensitive to the inhibitory effect of Nucant than SW480. This difference might be due to the fact that SW480 cells form larger clusters of cells that proliferate in several thick layers compared to the metastatic SW620 cells.

**Table 6:** Nucant pseudopeptides inhibit proliferation of human colon carcinoma cells.

Cells	Nucant	Number of cells	% inhibition
SW480	None: Control	1,500,000	-
	N7: 10 μM	1,200,000	20%
	N7: 20 μM	900,000	40%
	N6L: 10 μM	800,000	47%
			
SW620	None: Control	1,800,000	-
	N7: 10 μM	1,000,000	44%
	N7: 20 μM	700,000	61%
	N6L: 10 μM	450,000	75%

Cells seeded in 6-well plates at 20,000 cells/ml in 2 ml culture medium were cultured in the absence (Control) or presence of N7 and N6L. Seven days after seeding, cells were trypsinized and counted. Nucant pseudopeptides were added only once 5 hours after seeding of cells. Consistently, N6L manifests at least 2-fold higher inhibitory activity compared to N7.

**Figure 6:**
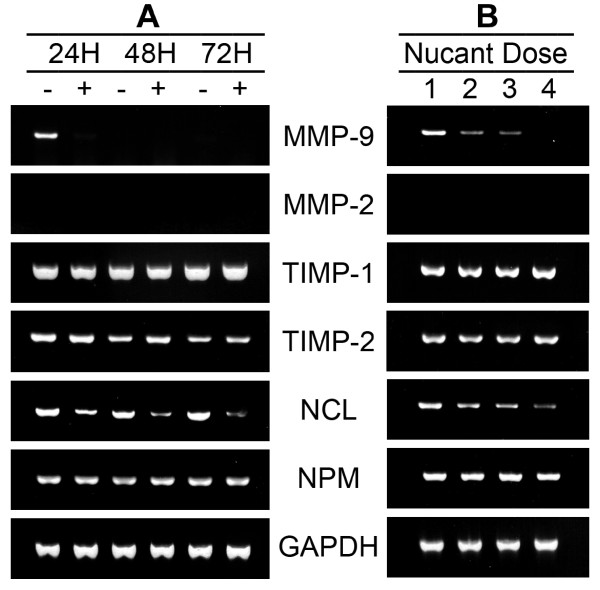
**Selective down regulation of MMP-9 and nucleolin transcripts in Nucant treated SW620 cells**. A. SW620 cells in the absence (lanes -) and presence (lanes +) of 10 μM of N7 were cultured for 24, 48, and 72 hours. B. Dose dependent reduction of MMP-9 and nucleolin transcripts in response to Nucant treatment. Newly seeded SW620 cells were cultured in the absence (lanes 1) and presence of 2.5 (lanes 2), 5 (lanes 3) and 10 (lanes 4) 10 μM of N6 for 20 hours. Total RNA extracts were prepared at different time points and the expression of various transcripts was analyzed by RT-PCR using specific primers (Methods). NCL stands for nucleolin; NPM stands for nucleophosmin.

The effect of Nucant on the expression of specific mRNAs was investigated in SW620 cells by RT-PCR using primers for: the matrix metalloproteinases MMP-2 and MMP-9 that in addition to modulation of the tumor microenvironment play an important role in cancer cell migration and even signaling pathways [71], the metalloproteinase inhibitors TIMP-1 and TIMP-2 [72], nucleolin that is induced constantly in proliferating tumor cells to generate surface nucleolin [15], nucleophosmin is another nucleolar protein that also interacts with Nucant [49], and the housekeeping gene GAPDH as a control. We found out that MMP-2 is not expressed in SW620 cells, whereas MMP-9 is expressed temporally and transiently, since it is expressed at 24 hours after passage of cells but drops completely at 48 hours (Figure 6A). Such variation and temporal expression of MMP-2 and MMP-9 mRNA in tumor cells has been reported previously [42,73]. The expression of the other genes, TIMP-1, TIMP-2, nucleolin, nucleophosmin, and GAPDH is readily detectable at different days after cell passage in SW620 cells. The results of a typical RT-PCR analysis are presented in Figure 6 showing the expression of various transcripts at 24, 48 and 72 hours after seeding of SW620 cells in the absence or presence of 10 μM of N7. Strikingly, Nucant treatment completely abolished the expression of MMP-9 transcripts observed at 24 hours post-seeding. In addition, Nucant treatment exerted a marked down regulation transcripts coding nucleolin at different days after passage of cells, whereas the expression of transcripts coding nucleophosmin was not affected (Figure 6A). Nucant mediated reduction of MMP-9 and nucleolin transcripts occurs by a dose-dependent manner and by a selective mechanism, since the expression of the other genes is not affected (Figure 6B). The molecular mechanism of such selective down regulation of MMP-9 mRNA might be the consequence, at least in part, of the marked down regulation of nucleolin. In this respect, it is of interest to note that nucleolin present in the cytoplasm binds 3'-untranslated region in MMP-9 mRNA, a process that is necessary for the stability and translational efficiency of MMP-9 mRNA [4]. Nucleolin-binding to MMP-9 mRNA increases the production of the enzyme that by degrading extracellular matrix components promotes tumor metastasis. Expression of MMP-9 is strongly linked with malignant tumor progression and metastasis of various types of cancers [71]. Similarly, expression of nucleolin is highly associated with tumor cell proliferation and angiogenesis [15]. Consequently, the selective down regulation of such strategic genes could account, at least in part, for the inhibitory mechanism on proliferation and spreading/migration of such colon carcinoma cells.

### Multiplication of T29 lymphoma cells is inhibited by the multivalent Nucant pseudopeptides due to a selective mechanism of cell death

In epithelial type tumor cell cultures, HB-19 and Nucant pseudopeptides could affect cell adhesion (Figure 4), migration (Additional file 5, Figure S7), spreading (Figure 5), and restore contact inhibition [38,42](Additional file 5, Figure S6), but have no significant effect on cell viability as demonstrated by the lack of trypan blue uptake in cells at 24 hours after treatment (Table 4, Figure 4B). In contrast to epithelial cells however, these pseudopeptides induce cell death in several leukemia cells (Table 4), in which nucleolin is highly expressed at the cell surface [15]. The binding and internalization of pseudopeptides in Leukemia cells result in a selective down regulation of surface but not nuclear nucleolin in a dose dependent manner (data not shown), as it is the case in epithelial cells (Figure 2B, D)[13,37].

**Figure 7:**
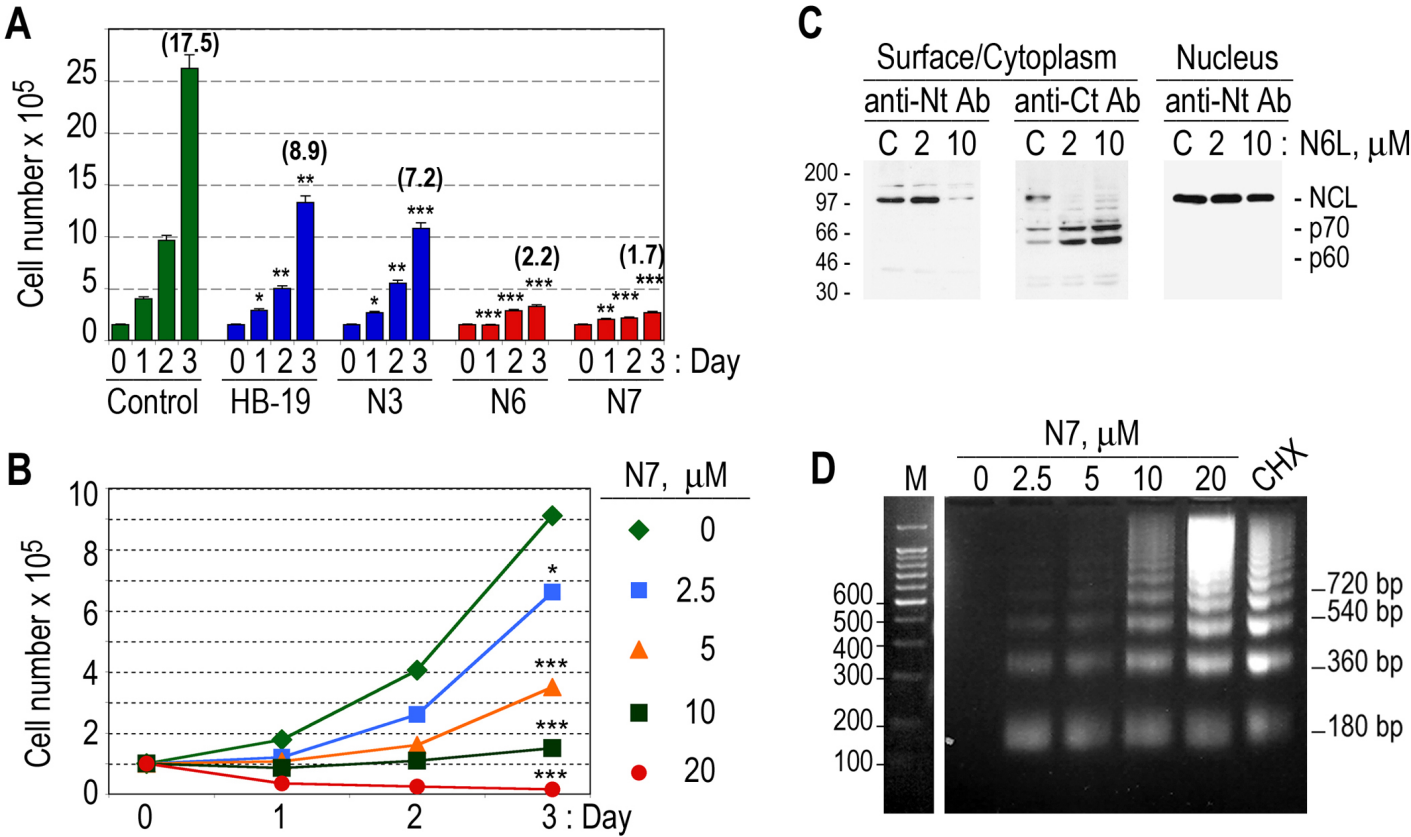
**HB-19 and related multivalent Nucant pseudopeptides induce cell death associated with internucleosomal DNA fragmentation**. A. Inhibition of cell multiplication by HB-19 and related multivalent Nucant pseudopeptides. T29 cells at 150,000 cells/ml in 2 ml of culture medium were seeded (Day 0) in the absence (Control) or presence of 20 μM of pentavalent (HB-19, N3) and 10 μM of hexavalent (N6 and N7) pseudopeptides. Cell cultures (in triplicate) were monitored for viable cell number daily for 3 days. The histograms show the mean number of cells/ml ± SD of triplicate samples. The multiplication index of cells at day 3 compared to day 0 (the day of seeding) is given at the top of histogram. At 3 days post passage of T29 cells, the cell multiplication index of cells is 17.5-fold compared to 8.9-, 7.2-, 2.2-, and 1.7-fold in the presence of treatment with HB-19, N3, N6, and N7, respectively. B. N7 inhibits T29 cell multiplication in a dose dependent manner. T29 cells seeded at 100,000/ml in 2 ml in the absence (0 μM) or presence of N7 at various concentrations 2.5, 5, 10 and 20 μM were cultured for 3 days and the number of viable cells was determined at day 1, 2, and 3 post-seeding. The ordinate gives the cell number/ml (the mean of 2 samples) in each culture at day 1, 2, and 3 post-seeding. C. Partial cleavage of surface/cytoplasmic nucleolin. T29 cells were cultured for 45 min with 0 (for control; lanes C), 2 and 10 μM of N6 before preparation of cytoplasmic (that contains both surface and cytoplasmic nucleolin) and nuclear extract and analysis by immunoblotting using rabbit polyclonal antibodies that are directed against the first 26 (panel anti-Nt Ab) and the last 16 amino acids (panel anti-Ct Ab) of human nucleolin, respectively. The nuclear extracts were analyzed by immunoblotting using anti-Nt Ab (Methods). The cleavage products, p60 and p70, are revealed with the anti-Ct Ab only. D. Internucleosomal DNA cleavage in Nucant treated Cells. T29 cells, untreated (lane 0) or treated at 2.5, 5, 10, and 20 μM of N6, or 10 μg/ml of cycloheximide (CHX) or 20 μM of bisphosphonate (BisP) for 24 hours, were processed for extraction of the low molecular weight DNA from the nucleoplasm (Methods). Lane M shows the electrophoresis mobility of the DNA marker composed of a 100 base-pair ladder. On the right is the position of nucleosomal fragments, starting from the monomer, dimer, trimer and tetramer unit of 180, 360, 540, and 720 bp, respectively. Statistical significance: *p < 0.1, **p < 0.01, ***p < 0.001.

Figure 7 presents a typical experiment in T29 lymphoma cells to demonstrate the inhibitory action of pentavalent (HB-19, N3) and hexavalent (N6 and N7) pseudopeptides on the proliferation of leukemia cells, T29 cells were passaged in the absence or presence of 20 μM and 10 μM of pentavalent and hexavalent pseudopeptides, respectively, and cell number was monitored daily for 3 days (Figure 7A). At 3 days post passage of T29 cells, the multiplication index of control cells is 17.5-fold compared to 8.9-, 7.2-, 2.2-, and 1.7-fold in the presence of treatment with HB-19, N3, N6, and N7, respectively. Thus the inhibitory activity of hexavalent pseudopeptides is stronger compared to that of pentavalent pseudopeptides, consistent with our previous observation in HeLa cells (Figure 2A). In order to estimate the 50% inhibitory concentration (IC50) values of the different pseudopeptides, we carried out several sets of experiments in which T29 cells were cultured for several days in the presence of 2.5, 5, 10, and 20 μM of each of the pentavalent and hexavalent pseudopeptides. A typical experiment is presented in Figure 7B showing that the reduction of cell number in response to treatment with HB-19 and Nucant pseudopeptides occurs in a dose dependent manner. More importantly, counting trypan blue positive cells, i.e. cells with permeable membranes, indicated that the reduction of leukemia cell number is in fact due to cell death. This latter is clearly demonstrated at high concentrations of pseudopeptide when viable cell number is reduced drastically as soon as 24 hours post-treatment, thus revealing the occurrence of cell death (for an example see Figure 7B at 20 μM of N7). Although there were some slight variations in the IC50 values from one experiment to the other, consistently the results indicated that the hexavalent pseudopeptides manifest several fold higher inhibitory activity compared to the pentavalent pseudopeptides. Table 7 gives the IC50 values for the various pseudopeptides in respect to their capacity to induce cell death and inhibit cell proliferation at 24 and 72 hours, respectively. Among the hexavalent Nucant pseudopeptides, N6L exerts 2 to 4 fold higher activity compared to that of its counterparts, N6 and N7. This is most probably due to its stable structure, since it presents the pseudo-tripeptide Lysψ(CH_2_N)-Pro-Arg all in L configuration (Table 1, Methods).

**Table 7:** IC50 values for the induction of cell death and reduction of cell number in T29 cells treated with HB-19 and related multivalent pseudopeptides.

Pseudopeptide (Multivalent)	Cell death: IC50 24 hours post-passage	Cell number: IC50 72 hours post-passage
HB-19 (pentavalent)	> 20 μM	20 μM
N3 (pentavalent)	> 20 μM	5 μM
N6 (hexavalent)	20 μM	8 μM
N7 (hexavalent)	20 μM	8 μM
N6L (hexavalent)	5-10 μM	2-4 μM

T29 cells were passaged in the presence of HB-19, N3, N6, N7, and N6L at various concentrations 0, 2.5, 5, 10 and 20 μM for 3 days. Twenty-four hours after the treatments, cell death was monitored by the uptake of trypan blue. The number of viable cells at 3 days post-treatment was used to estimate the IC50 values for the reduction of cell number. The pseudopeptides represent pentavalently or hexavalently the pseudo-tripeptide Lysψ(CH_2_N)-Pro-Arg (Figure 1).

Like in other cell types [15], surface/cytoplasmic nucleolin in T29 cells is degraded selectively upon treatment of cells with the Nucant pseudopeptides, since degradation of surface/cytoplasmic nucleolin occurs without any apparent effect on the level of nuclear nucleolin (Figure 7C). Interestingly, rabbit polyclonal antibodies directed against the last 16 but not the first 26 amino acid residues of nucleolin reveal the presence of partial cleavage products of 70- and 60-kDa (referred to as p70 and p60), thus indicating that such cleavage fragments are derived from the COOH-terminal end of nucleolin. Finally, we investigated whether Nucant-mediated cell death in leukemia cells is associated with internucleosomal DNA fragmentation (Figure 7D). No low molecular weight DNA fragments are observed in the nucleoplasm of control cells, whereas there is a Nucant-dose dependent increase of DNA fragments with a characteristic internucleosomal DNA cleavage ladder-pattern, generally observed in cells undergoing programmed cell death (PCD). A similar profile of DNA fragments was observed in cells treated with the anti-cancer drug bisphosphonate or the protein synthesis inhibitor cycloheximide [74], which are known to induce apoptosis.

**Figure 8:**
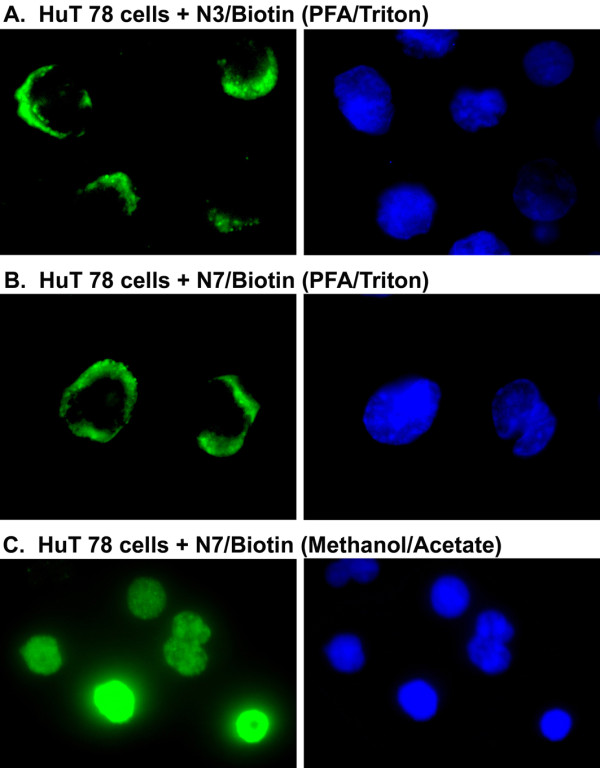
**Nucant pseudopeptides are internalized into cells and accumulate in the cytoplasm without translocation to the nucleus**. A/B. HuT 78 cells in polylysine-coated eight-well glass slides were incubated (37°C) with 15 μM of biotinylated N3 or N7 for 5 hours before PFA/Triton fixation and processing for immunofluorescence microscopy using rabbit anti-biotin and FITC conjugated goat anti-rabbit IgG. C. HuT 78 cells in polylysine-coated slides were incubated (37°C) with 15 μM of biotinylated N7 for 5 hours before methanol/acetate fixation and processing for immunofluorescence microscopy as in A/B (Methods). The nuclei stained with DAPI are shown on the right of each panel. Treatment of HuT 78 cells at 15 μM of biotinylated N3 or N7 leads to 25 and 40% cell death after 24 of culture, respectively.

In contrast to our results observed in MDA-MB 231 cells (Additional file 4, Figure S5), Nucant pseudopeptides were recently reported to be internalized and translocated into the nuclei of MDA-MB 231 cells [49]. In view of this and of the Nucant-induced cell death in leukemia cells, we investigated the entry of biotinylated N3 and N7 in the human leukemia HuT 78 cells. For this purpose HuT 78 cells were incubated (37°C, 5 hours) with 15 μM concentration of the biotinylated N3 and N7 construct, before PFA/Triton fixation and processing for immunofluorescence microscopy. Figure 8A and 8B indicate that most of the signal is located in the cytoplasm, with almost no signal in the nuclei. Consequently, we believe that the reported nuclear translocation of Nucant [49] is an artifact due to the experimental conditions that were used to fix cells. Indeed, Destouches et al have fixed cells with methanol/acetone, which appear to permeabilize the nuclear membrane and modify localization of Nucant during fixation of cells. Accordingly, when biotinylated N7 treated HuT cells were fixed with methanol/acetate, and then the entire signal was found to be localized in the nuclei of cells (Figure 8C).

The fact that Nucant does not affect cell viability in epithelial tumor cells indicates that the capacity of Nucant to induce cell death in leukemia cells is by a selective mechanism rather than due to non-specific cellular injury. The pathway by which Nucant induces cell death and the type of PCD remains to be investigated. The early loss of plasma membrane integrity in different types of leukemia cells in response to Nucant treatment favors necrotic type of PCD, since plasma membrane integrity is preserved during PCD by apoptosis in spite of internucleosomal DNA fragmentation [51,75]. Intriguingly, Nucant induced early loss of plasma membrane integrity is associated with internucleosomal cleavage of cellular DNA with the characteristic ladder pattern, which is generally observed in cells undergoing apoptosis. Previously, internucleosomal DNA cleavage, visualized as ladders, has been reported during necrosis in various types of cells, such as hepatocytes, thymocytes, Jurkat and MDCK cells [76].

### Nucant pseudopeptides bind surface nucleolin at a higher affinity compared to binding to nucleophosmin

**Figure 9:**
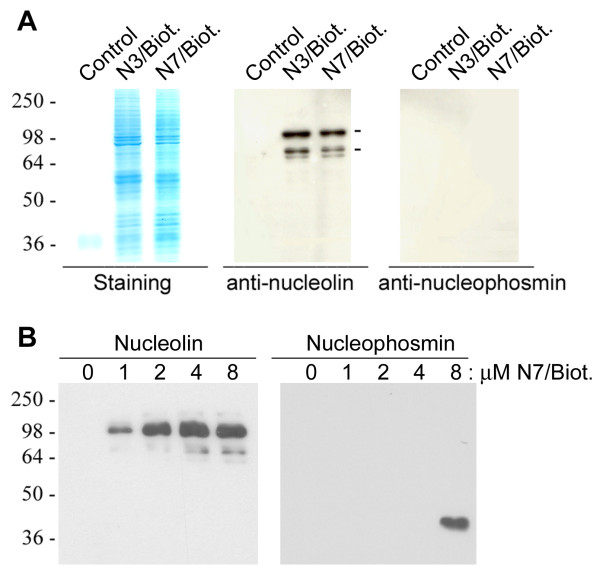
**Nucant pseudopeptides bind surface nucleolin at a higher affinity compared to binding to nucleophosmin**. A. Recovery of surface nucleolin and associated proteins by using biotinylated Nucant. MDA-MB 231 cells were incubated in the absence (lanes Control) or presence of 6 μM biotinylated N3 (N3/Biot.) or 4 μM of N7 (N7/Biot.) for the recovery of surface nucleolin and associated proteins under similar experimental conditions as described in the case of HB-19/Biot. (Methods). Purified material was then analyzed by SDS-PAGE and stained with Brilliant Blue G-Colloidal Concentrate staining (panel Staining). Similar samples were also analyzed by immunoblotting using anti-nucleolin mAb D3 (panel anti-nucleolin) or anti-nucleophosmin mAb EP1848Y (panel anti-nucleophosmin)(Methods). B. Nucant binds nucleophosmin independently of its capacity to bind to surface nucleolin. The T-cell leukemia HuT 78 cells were incubated at 0, 1, 2, 4, and 8 μM of the biotinylated N7 for the recovery of surface nucleolin and associated proteins. Nucleolin and nucleophosmin were revealed by immunoblotting as above.

Recently, by using the biotinylated Nucant construct, N6L, Destouches et al have reported that the recovery the cell surface nucleolin results in the copurification of several other proteins, including SRP68, SRP72, ribosomal proteins, and nucleophosmin [49]. As we did not recover nucleophosmin in our initial experiments using the biotinylated HB-19 (Table 2 and 3), two biotinylated Nucant constructs were used here for the recovery of surface nucleolin and nucleolin-associated proteins: the pentavalent N3 pseudopeptide that has a similar Aib contain template as N6L, and the hexavalent N7 pseudopeptide that has a similar template as HB-19 (Table 1). We first demonstrated that maximum binding to the surface nucleolin on MDA-MB 231 cells occurred at 5 and 3 μM concentration of the biotinylated N3 and N7, respectively (data not presented). Material purified from the surface of such cells was then analyzed by SDS-PAGE, before staining the pattern of the purified proteins and immunoblotting using anti-nucleolin or anti-nucleophosmin antibodies (Figure 9A). Both biotinylated N3 and N7 pseudopeptides resulted in the recovery of a similar pattern of proteins, including nucleolin as expected but not nucleophosmin. In another experiment we used T cell leukemia HuT 78 cells in which the Nucant pseudopeptide induces cell death (Table 4). As expected, the biotinylated N7 was found to bind nucleolin expressed on the surface of HuT 78 cells in a dose dependent manner, with maximum binding at 4 μM concentration. On the other hand, nucleophosmin binding occurred only at the highest concentration of the biotinylated N7 pseudopeptide (at 8 μM concentration; Figure 9B). These results indicate that Nucant binds nucleophosmin independently of its capacity to bind to surface nucleolin. Moreover, the binding affinity of Nucant to nucleophosmin is much lower compared to its binding affinity to surface nucleolin. These observations suggest that nucleophosmin is not one of the protein components of the 500-kDa complex. Consistent with this, Nucant is shown to bind nucleophosmin in solution [49]. Consequently, nucleophosmin represents an additional target of Nucant pseudopeptides (Table 8).

**Table 8:** The capacity of HB-19 and related multivalent Nucant pseudopeptides to bind ANP32A^1^, SET^1^, and nucleophosmin^2 ^expressed on the cell surface of cells in addition to nucleolin.

**ANP32A: **The acidic nuclear phosphoprotein 32 family, member A. In the literature it has also been referred to as LANP, MAPM, PP32, PHAP I, I1PP2A, C15orf1, MGC119787, and MGC150373. ANP32 is a 30-kDa phosphoprotein that is mainly described in the nucleus. It is characterized by an N-terminal tandem arrays of a leucine rich repeat and an acidic carboxyl half. ANP32A is implicated in a number of cellular processes, including modulation of cell signaling and transduction of gene expression to regulate the morphology and dynamics of the cytoskeleton, cell adhesion and differentiation, and caspase-dependent and caspase-independent apoptosis [78].

**SET nuclear oncogene**. In the literature it has also been referred to as 2PP2A, IGAAD, TAF-I, I2PP2A, IPP2A2, PHAP II, and TAF-IBETA. SET is a 39-kDa phosphoprotein with a highly acidic carboxyl-terminus. It is a multifunctional protein widely expressed in various tissues and localizes predominantly in the nucleus. It is involved in apoptosis, transcription, nucleosome assembly and histone binding [79].

**Nucleophosmin**. Also referred to as B23, nucleophosmin is a 37-kDa protein ubiquitously expressed chaperone that shuttles rapidly between the nucleus and cytoplasm, but predominantly resides in the nucleolus [101]. It is implicated in several cellular processes, including ribosome biogenesis, centrosome duplication, cell cycle progression, and apoptosis [77]. Somatic mutations in the exon 12 of the nucleophosmin gene (NPM1) are the most frequent genetic abnormality in adult acute myeloid leukemia leading to aberrant localization of nucleophosmin into the cytoplasm [77,101], which might be a critical event for leukogenesis [102].

**^1 ^**At concentrations ≥ 10 μM, HB-19 binds ANP32A (PHAP I) and SET (PHAP II) in addition to nucleolin expressed on the surface of cells [8].

**^2 ^**Nucant binds nucleophosmin at concentrations: ≥ 8 μM of N7 (in Figure 9), and at a concentration of N6L that was not stated in the article by Destouches et al [49].

Like nucleolin, nucleophosmin is a nucleo-cytoplasmic shuttling multifunctional protein with prominent nuclear localization. It is involved in many cellular processes, including the transport of pre-ribosomal particles and ribosome biogenesis, the response to stress stimuli, the maintenance of genomic stability, regulation of DNA transcription, and regulation of crucial tumor suppressors such as p53 and ARF [77]. The expression of nucleophosmin at the cell surface, and its implication in the overall mechanism of action of Nucant pseudopeptides against various tumor cell types remain to be characterized. Nevertheless, nucleophosmin and nucleolin expressed at the cell surface interact with K-Ras, and play a critical role in signal transduction via the MAPK pathway [25]. In general nucleophosmin appears to be relatively stable compared to the expression of surface nucleolin, which is constantly induced in association with the proliferative state of tumor cells [15].

## Conclusions

### HB-19 and related multivalent Nucant pseudopeptides are potent antitumoral agents by their capacity to exert multiple and distinct inhibitory effects

By their capacity to bind surface nucleolin in the 500-kDa protein complex, we show that HB-19 and related multivalent Nucant pseudopeptides exert multiple and distinct inhibitory effects on cell proliferation, adhesion, spreading, inflammation, and cell death. This is a unique property of HB-19 and related Nucant pseudopeptides, since other antitumoral agents do not exert a differential mode of action depending on a given tumor cell type. In addition to surface nucleolin, HB-19 and related Nucant pseudopeptides at high concentrations can bind directly other proteins expressed on the cell-surface, such as nucleophosmin [49] and the putative HLA class II-associated protein PHAP I and PHAP II [8](Table 8).

The official name of PHAP I and PHAP II is ANP32A for the acidic nuclear phosphoprotein 32 family member A, and the SET nuclear oncogene, respectively. Both ANP32A and SET have been described by several groups who have named them according to a specific function (Table 8). Like nucleolin, ANP32A and SET are nucleo-cytoplasmic shuttling phosphoproteins with various functions in cell metabolism. The common features between ANP32A and SET are the presence of an acidic carboxyl-terminal tail, association with HLA class II molecules, protein phosphatase 2 inhibitory activity, histone acetyltransferase inhibitory activity, and implication in mechanisms initiating cell death [78-81]. Consequently, Nucant-mediated occurrence of cell death in leukemia cells might be associated, at least in part, with the functioning of ANP32A and SET. In this respect, it is worthwhile to note that cross-linking of HLA class II has been reported to induce caspase independent cell death in lymphocytes [82]. Moreover, engagement of class II molecules mediates the transduction of signals leading to cell death, which is associated with the enhanced expression of IL-1β and TNF-α mRNA [83,84].

Several reports have now provided evidence that surface nucleolin is a promising target for cancer therapy [37,38,40,85,86]. Chemotherapy by targeting surface nucleolin could be less toxic compared to conventional cancer drugs, since nucleolin is continuously and abundantly expressed in tumor compared to normal cells, thus making tumor cells the preferential targets of inhibitors of surface nucleolin [15]. Another parameter that could contribute to the lack of toxicity is the capacity of HB-19 and related Nucant pseudopeptides to block the functioning of surface nucleolin without affecting nuclear nucleolin, which controls many aspects of cellular metabolism [1,3,15,37,38]. Indeed, after specifically binding to surface nucleolin, HB-19 and related Nucant pseudopeptides enter cells and accumulate in the cytoplasm without crossing the nuclear membrane. Consequently, the effect of these nucleolin antagonists is exerted differentially via the cell surface expressed nucleolin without affecting nuclear nucleolin.

The fact that nucleolin has several protein partners at the cell-surface, and the capacity of HB-19 and related Nucant pseudopeptides to bind additional cell surface proteins besides nucleolin, suggest that the response of tumor cells to these multivalent pseudopeptides should be associated with the expression and/or the level of surface nucleolin and the different nucleolin-partners in tumor cells. Consequently, these surface nucleolin antagonist pseudopeptides exert distinct inhibitory mechanisms depending on a given tumor cell type. Taken together, our results indicate that HB-19 and related Nucant pseudopeptides represent a unique multi-action drug, which provides novel therapeutic opportunities in treatment of a wide variety of cancers and related malignancies.

## Abbreviations

HB-19: the surface-nucleolin antagonist-pseudopeptide that presents pentavalently the tripeptide Kψ(CH_2_N)PR; HB-19/Btn: biotinylated HB-19; Nucant: nucleolin antagonist pseudopeptide that presents the tripeptide Kψ(CH_2_N)PR either pentavalently or hexavalently; N: Nucant; N3, HB-19 related pseudopeptide that presents pentavalently the tripeptide Kψ(CH_2_N)PR; N6/N6L/N7: HB-19 related pseudopeptides that present hexavalently the tripeptide Kψ(CH_2_N)PR; mAb: monoclonal antibody; MMP-2: matrix metalloproteinase-2; MMP-9: matrix metalloproteinase-9; TIMP-1: tissue inhibitor of metalloproteinase 1; TIMP-2: tissue inhibitor of metalloproteinase 2; GAPDH: glyceraldehyde-3-phosphate dehydrogenase; PCD: programmed cell death; PHAP: putative HLA class II-associated protein; ANP32A: acidic nuclear phosphoprotein 32 family member A.

## Competing interests

The authors declare that they have no competing interests.

## Authors' contributions

BK designed and performed experiments, and helped to draft the manuscript. DEK performed experiments, and helped to draft the manuscript. IN performed experiments. CS performed experiments. AGH conceived the overall research plan, designed and coordinated the various experiments, and wrote the paper. All authors read and approved the final manuscript.

## Pre-publication history

The pre-publication history for this paper can be accessed here:

http://www.biomedcentral.com/1471-2407/11/333/prepub

## Supplementary Material

Additional file 1 **The molecular structure of Nucant pseudopeptides, N3, N6, and N7**. N3 and N6 respectively present the pseudo-tripeptide Lysψ(CH_2_N)-Pro-Arg pentavalently and hexavalently, they are coupled to a polypeptide template containing Aib. N7 presents hexavalently the pseudo-tripeptide Lysψ(CH_2_N)-Pro-Arg coupled to a template similar to that of HB-19 (Figure S1). Click here for file

Additional file 2 **PAGE-SDS analysis of proteins associated with the cell surface expressed nucleolin in the 500-kDa complex**. The capacity of HB-19 to bind specifically surface nucleolin provides a convenient method to recover the nucleolin associated proteins, which were identified by microsequencing of their NH_2_-terminal ends (Figure S2). Click here for file

Additional file 3 **A potential candidate for a transmembrane protein partner of the cell surface expressed nucleolin is the low-density lipoprotein (LDL) receptor related protein (LRP1)**. In Chinese hamster ovary CHO LRP1-null cells, although nucleolin is present abundantly in the nucleus and in the cytoplasm, it remains undetectable at the cell surface (Figure S3). Consequently, in the absence of surface nucleolin in such LRP1-null cells, ligands of nucleolin are internalized by a receptor-independent passive process (Figure S4). Click here for file

Additional file 4 **Nucant pseudopeptides do not translocate into the nucleus**. Like other surface nucleolin ligands, HB-19 and related Nucant pseudopeptides enter cells by an active process and become accumulated in the cytoplasm without translocating to the nuclei (Figure S5). The recent report on nuclear translocation of N6L might be an artifact due to the experimental conditions in which cells were fixed with methanol/acetone. Click here for file

Additional file 5 **N6L treatment restores contact inhibition and reduces the motility of human breast cancer cells**. Treatment of MDA-MB 435 cells with Nucant leads to restoration of contact inhibition, while corresponding control tumor cells proliferate without contact inhibition by piling up over each other (Figure S6). In a wound-healing assay, invasion of the scratched area occurs much more freely in the untreated compared to N6L treated cell culture (Figure S7). Click here for file
