# Supplementary material for: Targeting surface nucleolin with multivalent HB-19 and related Nucant pseudopeptides results in distinct inhibitory mechanisms depending on the malignant tumor cell type
Source: BMC Cancer. 2011 Aug 3;11:333. doi: 10.1186/1471-2407-11-333 (PMC3199867; doi:10.1186/1471-2407-11-333)
Supplement: Additional file 1 — The molecular structure of Nucant pseudopeptides, N3, N6, and N7. N3 and N6 respectively present the pseudo-tripeptide Lysψ(CH2N)-Pro-Arg pentavalently and hexavalently, they are coupled to a polypeptide template containing Aib. N7 presents hexavalently the pseudo-tripeptide Lysψ(CH2N)-Pro-Arg coupled to a template similar to that of HB-19 (Figure S1). [file 1471-2407-11-333-S1.DOC]

**Additional file 1**

**The molecular structure of Nucant pseudopeptides, N3, N6, and N7.**

**Figure 1S.**

**NUCANT 3 (N3)** MW: 3249.15

**NUCANT 6 (N6)** MW: 3885.63

**NUCANT 7 (N7)** MW: 3709.7

N3 and N6 respectively present the pseudo-tripeptide Lysψ(CH_2_N)-Pro-Arg pentavalently and hexavalently, they are coupled to a polypeptide template containing Aib (2-aminoisobutyric acid). N7 presents hexavalently the pseudo-tripeptide Lysψ(CH_2_N)-Pro-Arg coupled to a template similar to that of HB-19 (see Table 1 and Methods in the Main text)[1]. The molecular structure of HB-19 and N6L has been reported previously [2-5].

1. Courty J, Hovanessian AG, Briand JP, Guichard G, Hamma-Kourbali Y: **Use of multivalent synthetic ligands of surface nucleolin for treating cancer or inflammation.** *WO 2007/125210 A2* 2007.

2. Nisole S, Krust B, Dam E, Blanco A, Seddiki N, Loaec S, Callebaut C, Guichard G, Muller S, Briand JP, Hovanessian AG: **The HB-19 pseudopeptide 5[Ky(CH_2_N)PR]-TASP inhibits attachment of T-lymphocyte- and macrophage-tropic HIV to permissive cells.** *AIDS Res Hum Retroviruses* 2000, **16**:237-249.

3. Destouches D, El Khoury D, Hamma-Kourbali Y, Krust B, Albanese P, Katsoris P, Guichard G, Briand JP, Courty J, Hovanessian AG: **Suppression of tumor growth and angiogenesis by a specific antagonist of the cell-surface expressed nucleolin.** *PLoS ONE* 2008, **3(6): e2518**.

4. Briand JP, Guichard G, Zimmer R: **New optically pure compounds for improved therapeutic efficiency.** *WO 2009/141687 A1* 2009.

5. Destouches D, Page N, Hamma-Kourbali Y, Machi V, Chaloin O, Frechault S, Birmpas C, Katsoris P, Beyrath J, Albanese P, Maurer, M, Carpentier, G, Strub, JM, Van Dorsselaer, A, Muller, S, Bagnard, D Briand, JP, Courty, J: **A Simple Approach to Cancer Therapy Afforded by Multivalent Pseudopeptides That Target Cell-Surface Nucleoproteins.** *Cancer Res* 2011, **71**(3296-3305).
