# Supplementary material for: Targeting surface nucleolin with multivalent HB-19 and related Nucant pseudopeptides results in distinct inhibitory mechanisms depending on the malignant tumor cell type
Source: BMC Cancer. 2011 Aug 3;11:333. doi: 10.1186/1471-2407-11-333 (PMC3199867; doi:10.1186/1471-2407-11-333)
Supplement: Additional file 2 — PAGE-SDS analysis of proteins associated with the cell surface expressed nucleolin in the 500-kDa complex. The capacity of HB-19 to bind specifically surface nucleolin provides a convenient method to recover the nucleolin associated proteins, which were identified by microsequencing of their NH2-terminal ends (Figure S2). [file 1471-2407-11-333-S2.DOC]

**Additional file 2**

**PAGE-SDS analysis of proteins associated with the cell surface expressed nucleolin in the 500-kDa complex.**

**Figure 2S. Surface nucleolin exists in a high molecular weight complex including proteins implicated in tumorigenesis, autoimmunity, and inflammation.**

**
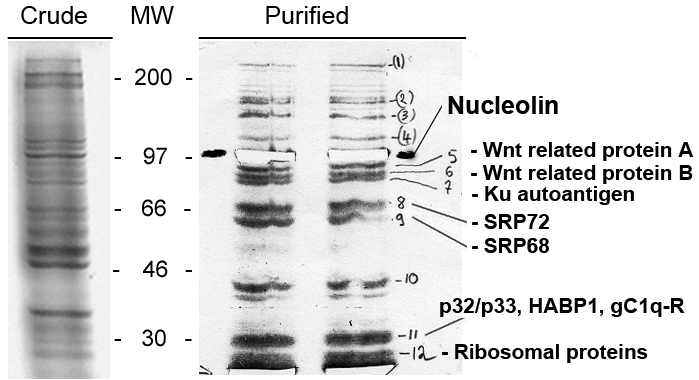
**

The 500-kDa complex containing surface nucleolin was purified from the cell surface by the capacity of HB-19 to bind specifically surface nucleolin [1-3] as described in the article. A small aliquot of crude extracts before purification (lane Crude) and two similarly purified samples (the two adjacent lanes Purified) were analyzed by SDS-PAGE (7.5%). The scan was taken after excision of the nucleolin bands at the electrophoresis mobility of 97-kDa. The numbers 1 to 12 show the other proteins that were recovered for microsequencing of the NH_2_-terminal ends. No sequence was obtained for proteins1-4 and 10, since their NH_2_-terminal ends seemed to be blocked. The other proteins were identified as it is indicated in the main text. Lane MW shows the profile of molecular weight protein markers.

1. Callebaut C, Jacotot E, Krust B, Guichar G, Blanco J, Svab J, Muller S, Briand JP, Hovanessian AG: **Pseudopeptides TASP inhibitors of HIV infection block viral entry by binding to a 95 kDa cell surface protein.** *J Biol Chem* 1997, **272**:7159-7166.

2. Nisole S, Krust B, Callebaut C, Guichard G, Muller S, Briand JP, Hovanessian AG: **The anti-HIV pseudopeptide HB-19 forms a complex with the cell-surface expressed nucleolin independent of heparan sulfate proteoglycans.** *J Biol Chem* 1999, **274**:27875-27884.

3. Nisole S, Said EA, Mische C, Prevost MC, Krust B, Bouvet P, Bianco A, Briand JP, Hovanessian AG: **The anti-HIV pentameric pseudopeptide HB-19 binds the C-terminal end of nucleolin and prevents anchorage of virus particles in the plasma membrane of target cells.** *J Biol Chem* 2002, **277**:20877-20886.
