# Supplementary material for: Targeting surface nucleolin with multivalent HB-19 and related Nucant pseudopeptides results in distinct inhibitory mechanisms depending on the malignant tumor cell type
Source: BMC Cancer. 2011 Aug 3;11:333. doi: 10.1186/1471-2407-11-333 (PMC3199867; doi:10.1186/1471-2407-11-333)
Supplement: Additional file 3 — A potential candidate for a transmembrane protein partner of the cell surface expressed nucleolin is the low-density lipoprotein (LDL) receptor related protein (LRP1). In Chinese hamster ovary CHO LRP1-null cells, although nucleolin is present abundantly in the nucleus and in the cytoplasm, it remains undetectable at the cell surface (Figure S3). Consequently, in the absence of surface nucleolin in such LRP1-null cells, ligands of nucleolin are internalized by a receptor-independent passive process (Figure S4). [file 1471-2407-11-333-S3.DOC]

**Additional file 3**

**A potential candidate for a transmembrane protein partner of the cell surface expressed nucleolin is the low-density lipoprotein (LDL) receptor related protein (LRP1).**

**Figure 3S. Lack of expression of nucleolin at the cell surface in LRP1-null cells.**


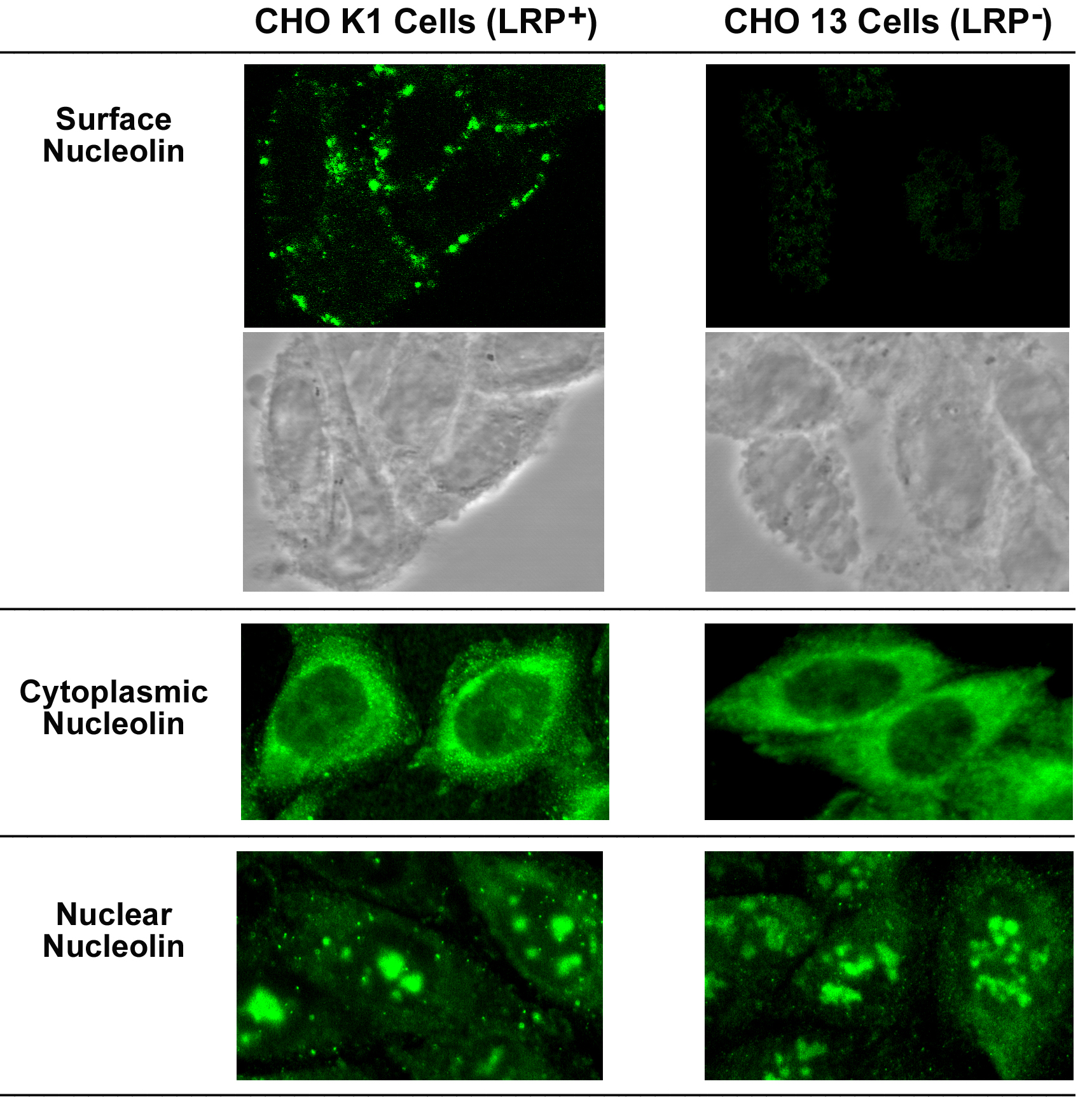


LRP-1 expressing CHO K1 and LRP-1 null CHO 13 cells in eight-well glass slides (Lab-Tek Brand; Nalge Nunc International, Naperville, IL) were plated 24 hours before the experiment. Cells were fixed with either 3.7% paraformaldehyde (10 min) for membrane staining to monitor the clustered surface nucleolin, or 7% PFA to stain cytoplasmic nucleolin, or PFA/Triton X-100 (3.7%/ 0.5%) for staining of nuclear nucleolin [1, 2]. For labeling of surface nucleolin, cells were incubated (45 min, 8°C) with rabbit antiserum against hamster nucleolin before PFA fixation and processing for confocal laser immunofluorescence microscopy (a phase contrast image of cells is also presented). Incubation at room temperature prevents intracellular translocation of antibody-nucleolin complex and allows antibody-dependent clustering of surface nucleolin. 7% PFA fixation permeabilizes the plasma but not the nuclear membrane allowing staining of cytoplasmic nucleolin in the absence of the strong signal of nuclear nucleolin. The secondary antibody is a FITC-conjugated goat anti-rabbit IgG (Sigma). All the experimental conditions for confocal immunofluorescence microscopy were as described previously [1].

The results show that surface nucleolin is detectable in CHO K1 but not in CHO 13 cells, although in both cell types nucleolin is expressed abundantly in the cytoplasm and in the nucleus. These results suggest that surface nucleolin expression is dependent on the expression of LRP-1, the mechanism of which remains to be elucidated.

**Figure 4S. Passive entry of a surface nucleolin ligand in LRP-1 null cells in contrast to the conventional active entry process in LRP-1 expressing cells.**


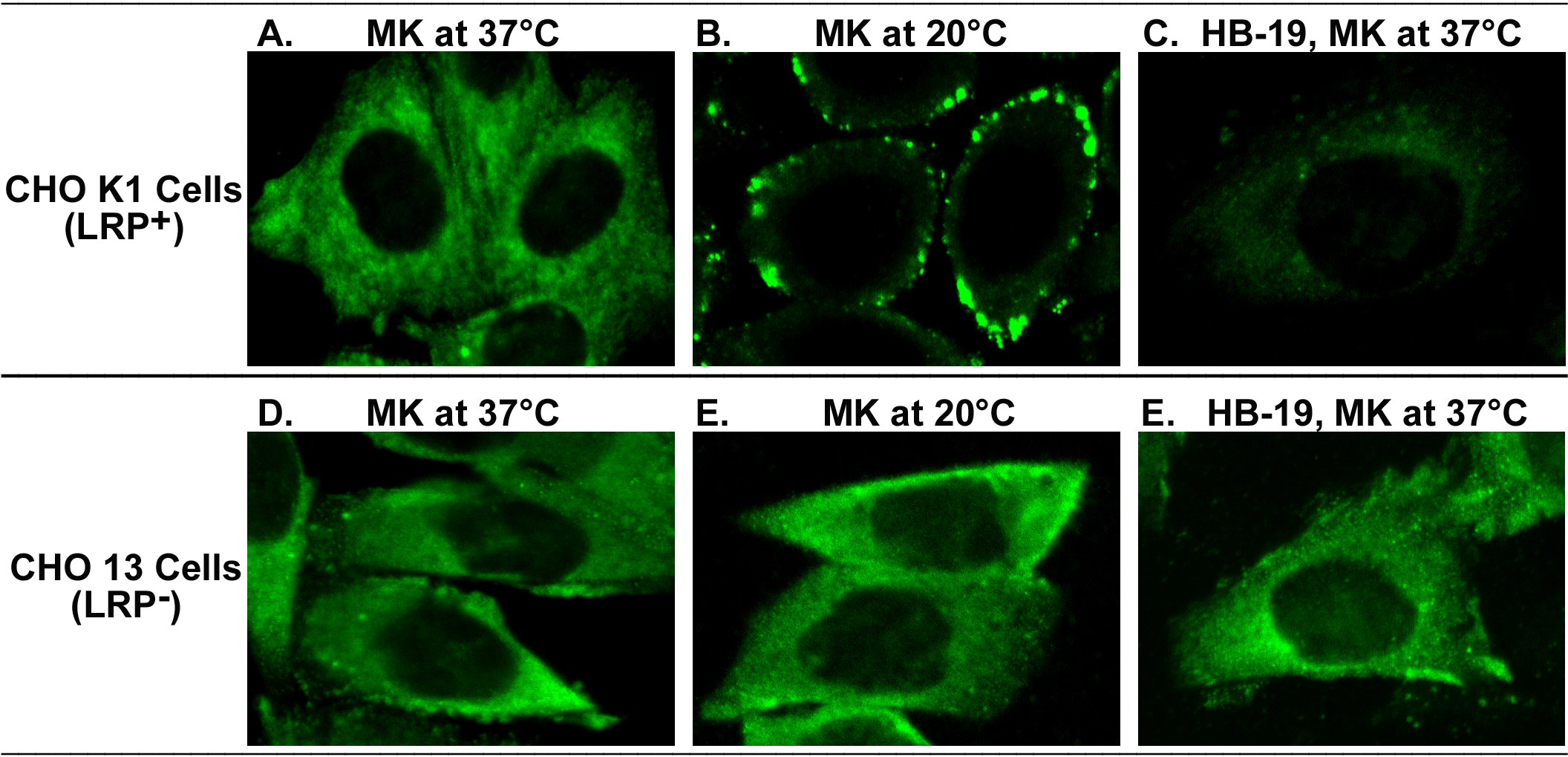


CHO K1 and CHO 13 cells (as in the Additional file 2, Figure 3S) were incubated with 0.2 μM Midkine (MK) for 90 min at 37°C or at 20°C before fixation with PFA. In sections C and E, cells were first incubated (30 min at 20°C) with 5 μM HB-19 before addition of MK (0.2 μM) and further incubation at 37°C for 90 min. The primary antibody was goat anti-MK that was revealed by FITC conjugated rabbit anti-goat antibody. Experimental conditions for confocal immunofluorescence microscopy were as described previously [1].

The results show that in LRP**^+ve^**/surface nucleolin**^+ve^** CHO K1 cells MK internalization occurs at 37 but not at 20°C thus indicating that MK entry is by an active process (sections A and B), which requires binding to the RGG domain of surface nucleolin. Accordingly, pretreatment of K1 cells with HB-19 that inhibits the binding of MK to cells [3, 4] prevents MK entry (section C). On the other hand, MK entry in LRP**^-ve^**/surface nucleolin**^-ve^** CHO 13 cells occurs by a passive mechanism, since it occurs both at 37 and 20°C (sections D and E) and is not affected by HB-19 pretreatment (section F). Therefore, in the absence of surface nucleolin, MK enters freely into the cytoplasm. It is worthwhile to note that as the internalization surface nucleolin ligands occurs through lipid rafts, destruction of membrane cholesterol in various types of cells results in internalization of such ligands by a receptor-independent passive process [4, 5](Unpublished data).

1. Hovanessian AG, Puvion-Dutilleul F, Nisole S, Svab J, Perret E, Deng JS, Krust B: **The cell-surface-expressed nucleolin is associated with the actin cytoskeleton.** *Exp Cell Res* 2000, **261**:312-328.

2. Hovanessian AG, Soundaramourty C, El Khoury D, Nondier I, Svab J, Krust B: **Surface expressed nucleolin is constantly induced in tumor cells to mediate calcium-dependent ligand internalization.** *PLoS One* 2010, **5(12):e15787**.

3. Callebaut C, Nisole S, Briand JP, Krust B, Hovanessian AG: **Inhibition of HIV infection by the cytokine midkine.** *Virology* 2001, **281**:248-264.

4. Said AE, Krust B, Nisole S, Briand JP, Hovanessian AG: **The anti-HIV cytokine midkine binds the cell-surface-expressed nucleolin as a low affinity receptor**. *J Biol Chem* 2002, **277**:37492-37502.

5. Said EA, Courty J, Svab J, Delbé J, Krust B, Hovanessian AG: **Pleiotrophin inhibits HIV infection by binding the cell surface expressed nucleolin.** *FEBS J* 2005, **272**:4646-4659.
