# Supplementary material for: Targeting surface nucleolin with multivalent HB-19 and related Nucant pseudopeptides results in distinct inhibitory mechanisms depending on the malignant tumor cell type
Source: BMC Cancer. 2011 Aug 3;11:333. doi: 10.1186/1471-2407-11-333 (PMC3199867; doi:10.1186/1471-2407-11-333)
Supplement: Additional file 4 — Nucant pseudopeptides do not translocate into the nucleus. Like other surface nucleolin ligands, HB-19 and related Nucant pseudopeptides enter cells by an active process and become accumulated in the cytoplasm without translocating to the nuclei (Figure S5). The recent report on nuclear translocation of N6L might be an artifact due to the experimental conditions in which cells were fixed with methanol/acetone. [file 1471-2407-11-333-S4.DOC]

**Additional file 4**

**Nucant pseudopeptides do not translocate into the nucleus.**

**Figure 5S. Entry of HB-19 and related Nucant pseudopeptides into the cytoplasm but not into the nucleus of human breast cancer cells.**


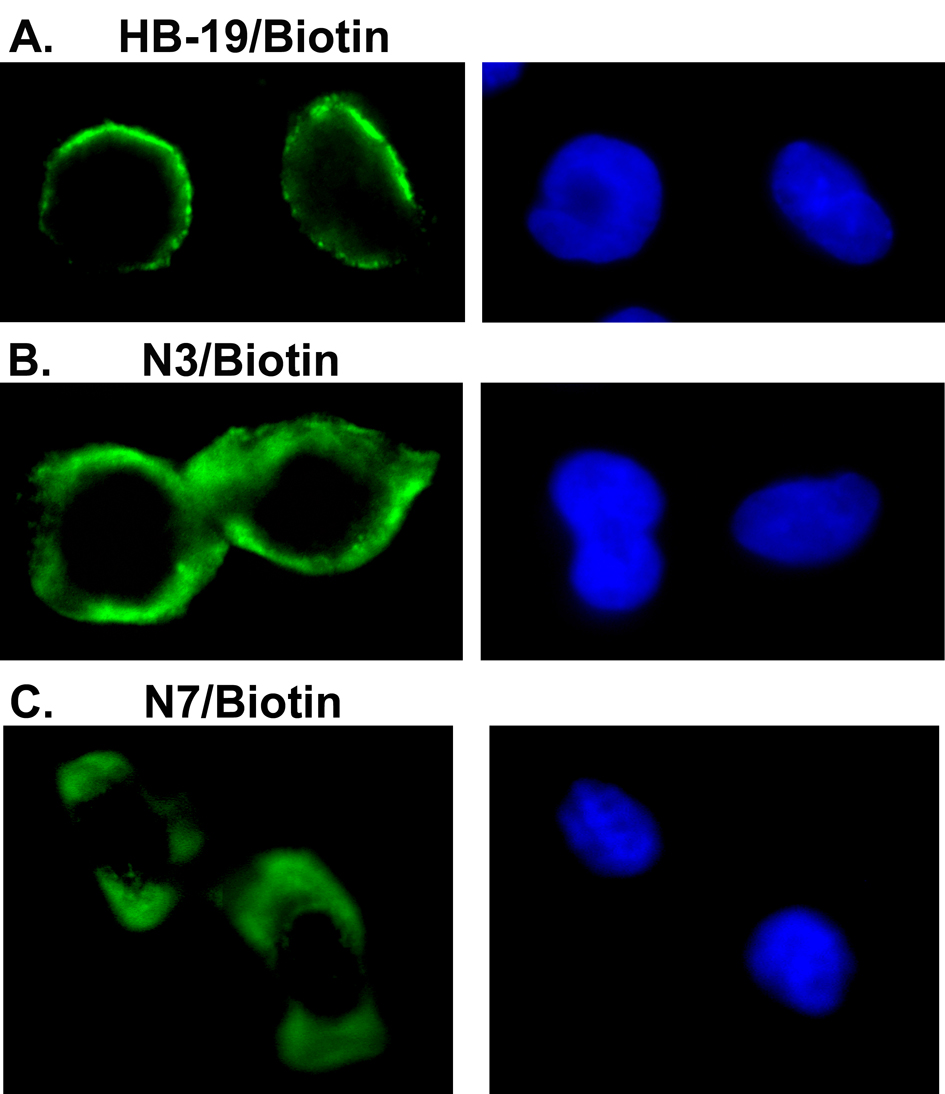


Subconfluent MDA-MB 231 cells plated in eight-well glass slides were incubated (37°C, 5 hours) with biotinylated HB-19, N3 or N7 (each at 10 μM concentration). PFA/Triton fixed cells were then processed for immunofluorescence microscopy using rabbit anti-biotin and FITC conjugated goat anti-rabbit IgG. Nuclei were colored with DAPI.

Like other surface nucleolin ligands, HB-19 and related Nucant pseudopeptides enter cells by an active process and become accumulated in the cytoplasm without translocating to the nuclei ([1-5]; and unpublished data). In these studies cells were fixed with PFA/Triton before confocal laser immunofuorescence microscopy. The lack of Nucant entry into the nuclei was also observed in PFA/Triton fixed leukemia cells in which Nucant results in cell killing as soon as 16-24 hours after treatment (see Main text). In such leukemia cells, biotinylated N3 or N7 enter the cytoplasm but they do not translocate into the nuclei of cells (Main text, Figure 8). In contrast to these observations, N6L has been claimed to enter the nuclei of MDA-MB 231 cells [6]. We believe that this latter is an artifact due to the experimental conditions that were used to fix cells with methanol/acetone, which appear to permeabilize the nuclear membrane and modify localization of Nucant during fixation of cells. Moreover, a strong fluorescent N6L signal in the cytoplasm could falsely be interpreted as a nuclear signal, since detection of immunofluorescence was carried out simply by using a fluorescence microscope [6]. Fixation of cells with PFA/Triton and confocal laser immunofluorescence microscopy could have clearly demonstrated that the results obtained after methanol/acetone fixation are artifactual.

1. Said AE, Krust B, Nisole S, Briand JP, Hovanessian AG: **The anti-HIV cytokine midkine binds the cell-surface-expressed nucleolin as a low affinity receptor**. *J Biol Chem* 2002, **277**:37492-37502.

2. Said EA, Courty J, Svab J, Delbé J, Krust B, Hovanessian AG: **Pleiotrophin inhibits HIV infection by binding the cell surface expressed nucleolin.** *FEBS J* 2005, **272**:4646-4659.

3. Destouches D, El Khoury D, Hamma-Kourbali Y, Krust B, Albanese P, Katsoris P, Guichard G, Briand JP, Courty J, Hovanessian AG: **Suppression of tumor growth and angiogenesis by a specific antagonist of the cell-surface expressed nucleolin.** *PLoS ONE* 2008, **3(6): e2518**.

4. Legrand D, Vigie K, Said EA, Elass E, Masson M, Slomianny MC, Carpentier M, Briand JP, Mazurier J, Hovanessian AG: **Surface nucleolin participates in both the binding and endocytosis of lactoferrin in target cells.** *Eur J Biochem* 2004, **271**:303-317.

5. Hovanessian AG: **Midkine is a cytokine that inhibits HIV infection by binding to the cell surface expressed nucleolin.** *Cell Res* 2006, **16**:174-181.

6. Destouches D, Page N, Hamma-Kourbali Y, Machi V, Chaloin O, Frechault S, Birmpas C, Katsoris P, Beyrath J, Albanese P, Maurer, M, Carpentier, G, Strub, JM, Van Dorsselaer, A, Muller, S, Bagnard, D Briand, JP, Courty, J: **A Simple Approach to Cancer Therapy Afforded by Multivalent Pseudopeptides That Target Cell-Surface Nucleoproteins.** *Cancer Res* 2011, **71**(3296-3305).
