# Supplementary material for: Targeting surface nucleolin with multivalent HB-19 and related Nucant pseudopeptides results in distinct inhibitory mechanisms depending on the malignant tumor cell type
Source: BMC Cancer. 2011 Aug 3;11:333. doi: 10.1186/1471-2407-11-333 (PMC3199867; doi:10.1186/1471-2407-11-333)
Supplement: Additional file 5 — N6L treatment restores contact inhibition and reduces the motility of human breast cancer cells. Treatment of MDA-MB 435 cells with Nucant leads to restoration of contact inhibition, while corresponding control tumor cells proliferate without contact inhibition by piling up over each other (Figure S6). In a wound-healing assay, invasion of the scratched area occurs much more freely in the untreated compared to N6L treated cell culture (Figure S7). [file 1471-2407-11-333-S5.DOC]

**Additional file 5**

**N6L treatment restores contact inhibition and reduces the motility of human breast cancer cells.**

**Figure 6S. Nucant treatment of breast cancer cells restores contact inhibition.**


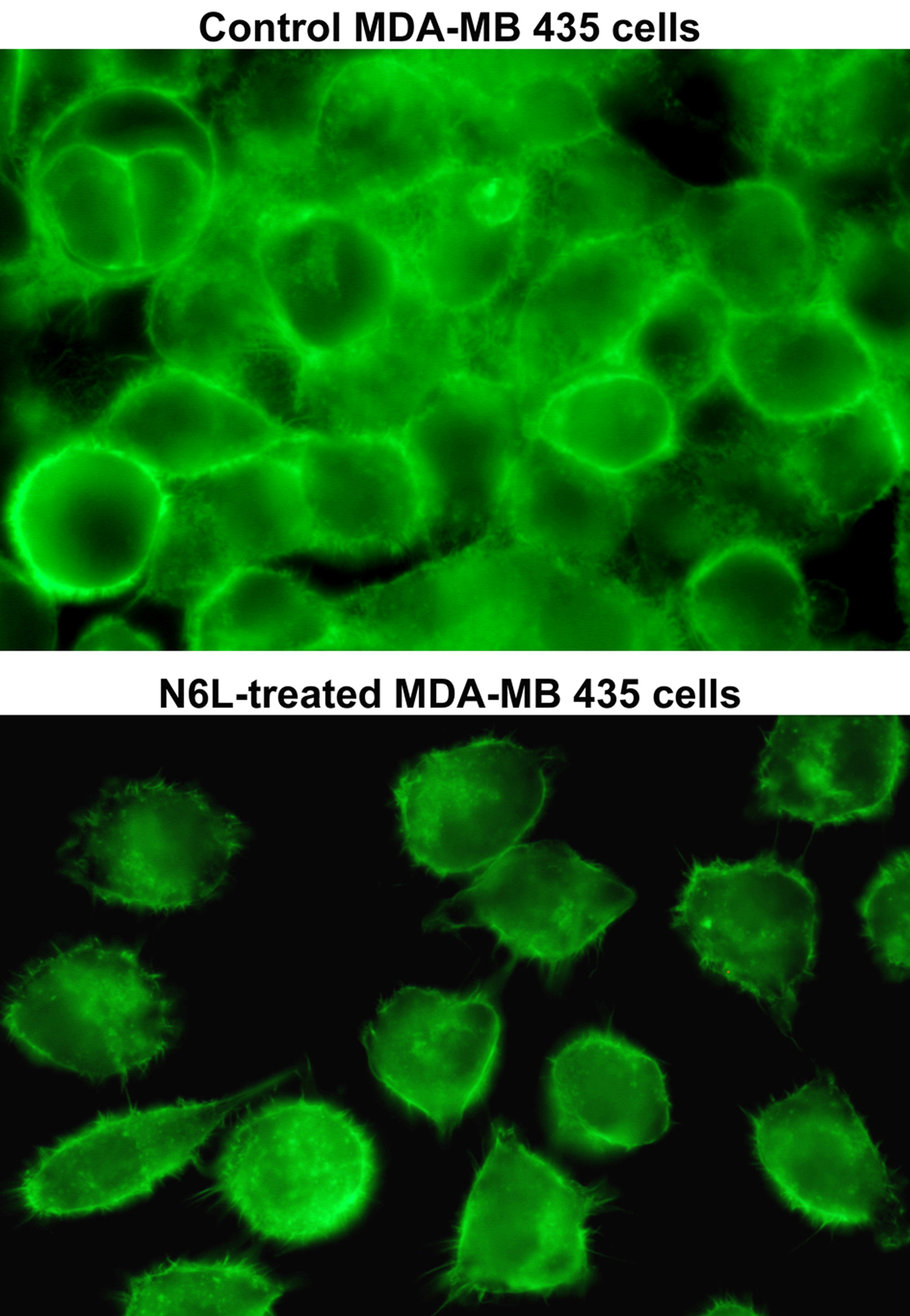


MDA-MB 435 cells plated in eight-well glass slides were cultured for 4 days in the absence (Control) or presence of N6L (20 μM). PFA/Triton fixed cells were then processed for immunofluorescence microscopy using mAb D3 against nucleolin [1].

The results demonstrate that control tumor cells proliferate without contact inhibition by piling up over each other, which is characteristic of migration and/or invasion of tumorigenic cells. On the other hand, there is a restoration of contact inhibition after 4 days of treatment with N6L. A similar restoration of contact inhibition was observed in rhabdoid tumor derived G401 cells that were cultured for several weeks at a 10-fold lower dose of HB-19 (2 μM) [2].

**Figure 7S. Nucant treatment reduces the motility of tumor cells.**


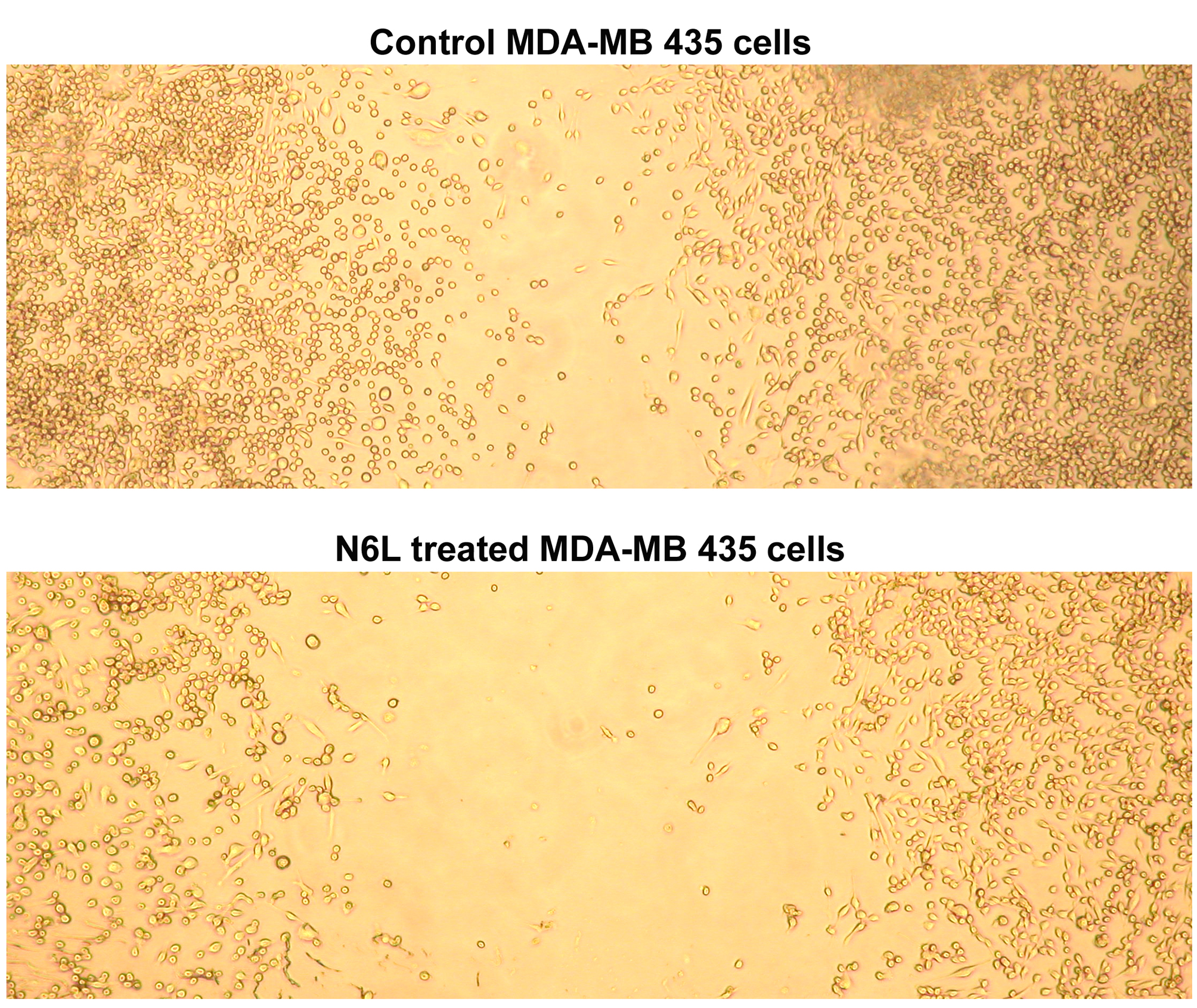


The wound healing assay of control and N6L treated MDA-MB 435 cells was performed as described previously [3]. Briefly, confluent monolayers of cells (plated onto 60 mm^2^ dishes) were scratched using 200 μl pipette tip, and cells were cultured in the absence (Control) or presence of N6L (10 μM) for 48 hours. Wound closure was monitored over time and photographed using Olympus 1-81 microscope at 40X magnification.

These results demonstrate that invasion of the scratched area occurs much more freely in the untreated compared N6L treated cell culture. Indeed, almost no cells are present at the middle of scratched area in N6L treated cells compared to the corresponding control cells.

1. Hovanessian AG, Puvion-Dutilleul F, Nisole S, Svab J, Perret E, Deng JS, Krust B: **The cell-surface-expressed nucleolin is associated with the actin cytoskeleton.** *Exp Cell Res* 2000, **261**:312-328.

2. Krust B, El Khoury D, Soundaramourty C, Nondier I, Hovanessian AG: **Suppression of tumorigenicity of rhabdoid tumor derived G401 cells by the multivalent HB-19 pseudopeptide that targets surface nucleolin.** *Biochimie* 2011, **93**:426-433.

3. Messi E, Florian MC, Caccia C, Zanisi M, Maggi R: **Retinoic acid reduces human neuroblastoma cell migration and invasiveness: effects on DCX, LISI, neurofilaments-68 and vimentin expression.** *BMC Cancer* 2008, **8:30**.
